# Regiospecific Cation Exchange in Nanocrystals and Its Potential in Diversifying the Nanostructural Library

**DOI:** 10.1002/smsc.202200063

**Published:** 2022-12-03

**Authors:** Yongju Hong, Sandhya Venkateshalu, Sangyeon Jeong, Jongsik Park, Kwangyeol Lee

**Affiliations:** ^1^ Department of Chemistry and Research Institute for Natural Sciences Korea University Seoul 02841 Republic of Korea; ^2^ Department of Chemistry Kyonggi University Suwon 16227 Republic of Korea

**Keywords:** anion exchange, cation exchange, copper sulfide, heterostructures, regiospecificity

## Abstract

The cation‐exchange reaction (CER), a promising nanocrystal (NC) engineering strategy, has undergone rapid progress in the past decade, sparking a big wave of interest in the post‐synthetic tuning of chemical compositions, crystal phases, interfaces, morphologies, and corresponding properties. However, a significant gap has existed between the theoretical and actual CERs, hindering the popularization of CERs for explosive expansion in NC designs. A notable roadblock in this area has been the inability to control the site of cation exchange within the nanostructure, although partial cation exchange at desired sites can open an avenue to the vast structural diversity of nanostructures and accompany new physicochemical properties. Several notable successes have been recorded recently in fabricating predesigned hetero‐nanostructures by thoroughly understanding the principles of cation exchange and by exploiting the peculiarity of each crystal system. Herein, recent advances achieved in the CER are introduced, unraveling the critical factors controlling regiospecificity by analyzing the developed theories and accumulated experimental results. It is further described how this knowledge can be harnessed to design advanced NCs, and the beneficial effect of regiospecificity on material properties is highlighted. Finally, the challenges and research directions are provided to encourage further research in this burgeoning field.

## Graphical abstract

Current challenges in cation‐engineered nanocrystals (NCs) have been addressed, emphasizing the importance of regiospecificity control during cation‐exchange reactions. In particular, a comprehensive list of studies on regiospecificity‐engineered hetero‐nanostructures is detailed, primarily focused on improving corresponding physicochemical properties, and the favorable effects of the geometrics are systematically organized based on the structures of NCs.

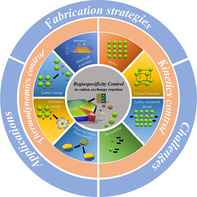

## 1 Introduction

Inorganic colloidal nanocrystals (NCs), by virtue of their tunable electronic and optical properties enabled by precisely controlled compositions and morphologies, have been at the forefront of research in chemistry and material science. In the past few decades, numerous strategies have been developed to directly synthesize inorganic NCs, such as hot injection,^[^ ^1^ ^]^ solvothermal,^[^ ^2^ ^]^ and hydrothermal methods.^[^ ^3^, ^4^ ^]^ As of today, scientists can prepare inorganic NCs of a wide variety of materials with structural precision matching that of molecules in some cases. There are enormous amounts of reports discussing the parameters that regulate the nucleation and growth of NCs, their shape, size, and interaction between the organic ligand molecules, and the specific surface of NCs.^[^ ^5^, ^6^ ^]^ Although impressive knowledge has been accumulated about synthetic routes and physicochemical properties of NCs, researchers still desire more effective synthetic strategies for achieving new functionalities and enhanced properties, which are always in demand. Synthetic approaches that produce NCs with multicomponent and interfaces are generally based on surface growth, templating, or nanoreactor fabrication methods, requiring specialized techniques.^[^ ^7^ ^]^ Furthermore, these methods produce NCs with limited complexity and are not applicable to all the systems, thus instigating the need to develop optimized synthetic pathways applicable to specific systems. In contrast, ion engineering can post‐synthetically modify diverse classes of readily available NCs without requiring specialized equipment. Intraparticle CER is a topotactic process that replaces cations in ionic NCs while preserving the intact anion framework. This convenient and facile post‐synthetic modification strategy can single‐handedly achieve requirements such as tuning compositions, phases, and interface structures.^[^ ^7^ ^]^ Therefore, considerable efforts have been devoted to designing sophisticated heterostructures of NCs by mediating the thermodynamics and kinetics of CERs, which are typically not accessible by direct synthetic methods.^[^ ^8^ ^]^

The formation of heterostructures not only integrates properties of two or more different materials in a single structure but also provides a means of influencing charge carrier separation and recombination, which is not available in single‐phase structures and may further promote other synergistic interactions between the distinctive domains.^[^ ^9^, ^10^ ^]^ The partial exchange reactions (CERs) involving the replacement of only a fraction of the cations are widely studied to produce heterostructured NCs.^[^ ^5^, ^7^, ^11^ ^]^ However, the resulting structures usually appear as heterostructures of arbitrary complexity. The design and synthesis of structurally well‐defined heterostructured NCs are limited by the lack of understanding of the regiospecificity.

Most cases of CER discussed in previous reports and literature reviews are not free of the shortcomings of random cation‐exchange sites.^[^ ^5^, ^8^, ^9^, ^12^ ^]^ However, the recent breakthroughs in CER enabled the thorough understanding of the cation‐exchange mechanism and thus the kinetically controlled partial CER, allowing for the delicate tuning of the NC properties. The synergistic interaction of two adjoined phases at the heterointerface, prepared by controlled partial CER, opens up a wide avenue to the development of new catalyst classes, which can unveil hitherto unobserved catalytic functions. Therefore, a report that systematically organizes various aspects of regiospecifically well‐designed CER would be timely and important.

The rational expansion of the current uncontrolled partial cation exchange would be the search for the methodology to induce the regiospecific movement of atoms within the NCs. The ability to locate ion‐specific sites would naturally lead to the direction‐controlled ion exchange within the NC, which would be a solution for nanotechnology to leap forward, facilitating the utilization of nanomaterials.

**Figure 1:**
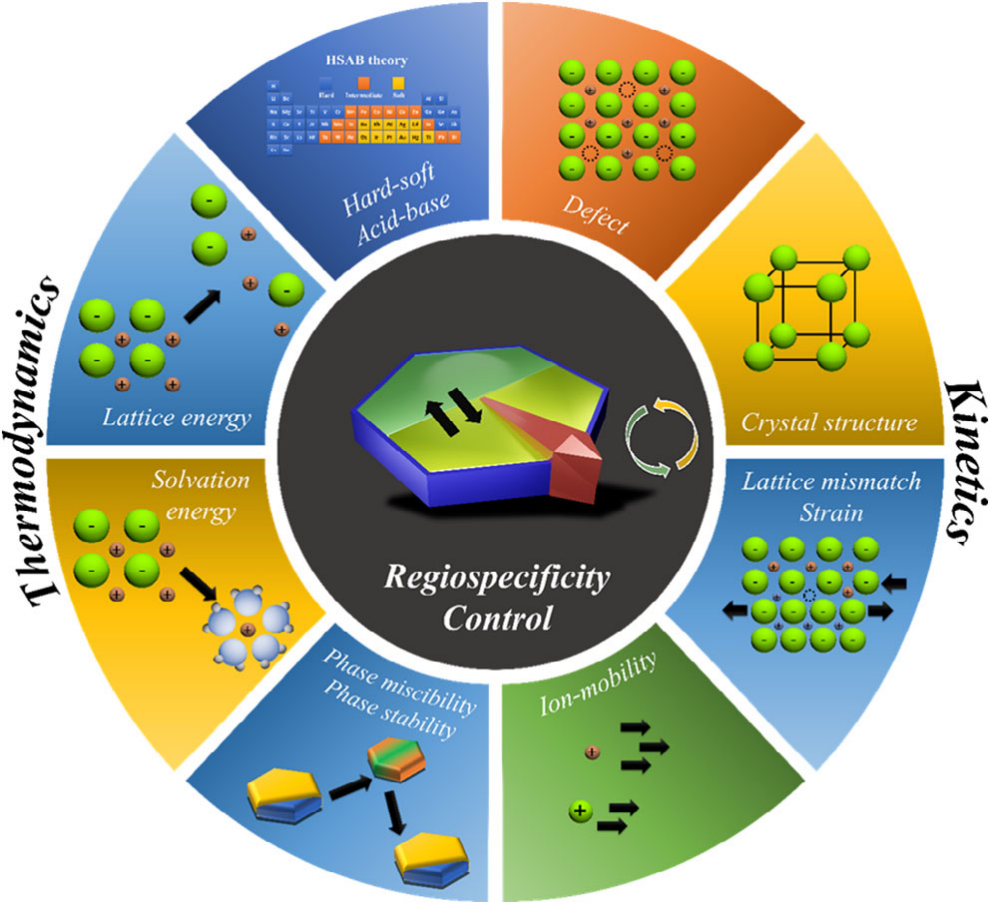
Schematic representing the factors influencing the regiospecificity control during the ion‐exchange reactions in the NCs.

This perspective showcases the advances in theoretical and experimental studies that have furthered our fundamental understanding of the regiospecific behavior of CERs. First, we introduce the concept of “regiospecificity,” including the principles of CER in NCs, by unearthing various reaction‐affecting parameters (**Figure** **1**). Then, we discuss recent progress in the regiospecific behavior of cations during the CERs, of which the composition and morphologies are precisely manipulated. Next, we review the latest trends in the synthesis, emphasizing the regiospecificity control and various experimental and theoretical studies correlating the optical and electrochemical properties. Finally, challenges associated with regiospecifically ion‐engineered NCs are discussed, and a perspective of the future research directions is provided.

## 2 Fundamental Understanding of Basic Principles and Rules of Design

The physicochemical properties of NCs can be finely tuned by their composition, size/shape, and heterointerfaces, making them promising candidate materials for many applications.^[^ ^13^, ^14^, ^15^, ^16^, ^17^, ^18^ ^]^ Ion exchange on the various template NCs available has been proposed as a promising strategy for heterostructured nanoparticle synthesis and attracted tremendous interest.^[^ ^7^, ^9^, ^11^ ^]^ This section mainly discusses fundamental principles and experimental insights into the rules of CERs for developing NCs.

A variety of reaction outcomes are possible through CER depending on the reaction conditions such as temperature, solvents, additives, etc. Careful consideration of synthetic parameters and chemical reactivity is required.^[^ ^6^ ^]^ Therefore, understanding thermodynamics and kinetics is the most important factor in inducing targeted CERs. Not surprisingly, many definitions exist to describe and quantify the observed ion‐exchange reactions in different conditions. In a given monovalent CER, the process can be simply denoted by Equation (1)

(1) $$MX_{\left(\text{crystals}\right)}+N_{\left(\text{sol}.\right)}^{m+}\to NX_{\left(\text{crystals}\right)}+M_{\left(\text{sol}.\right)}^{m+}$$

where *M* and *N* represent the metallic element while X represents the nonmetallic element. Specifically, the equations can be expanded to Equation (2), indicating redox reactions when the exchanging metals have heterovalent cations in unequal oxidation states.

(2) $$nM_{x}X_{m\left(\text{crystals}\right)}+mxN_{\left(\text{sol}.\right)}^{n+}\to mN_{x}X_{n\left(\text{crystals}\right)}+nxM_{\left(\text{sol}.\right)}^{m+}$$

According to the same treatment proposed by Rivest et al., the process can be divided into four subprocesses, and the spontaneity of ion‐exchange reaction can be quantified by calculating the Gibbs free energy (Δ*G*) (Equation (3)), where *E* ^0^ is the standard redox potential.^[^ ^19^ ^]^

(3) $$\Delta G_{\text{reaction}}=-m\Delta G_{\text{formation}}(N_{x}X_{n})-n\Delta G_{\text{formation}}\left(M_{x}X_{m}\right)+xF(nE_{N^{n+}/N}^{0}-mE_{M^{m+}/M}^{0})$$

**Figure 2:**
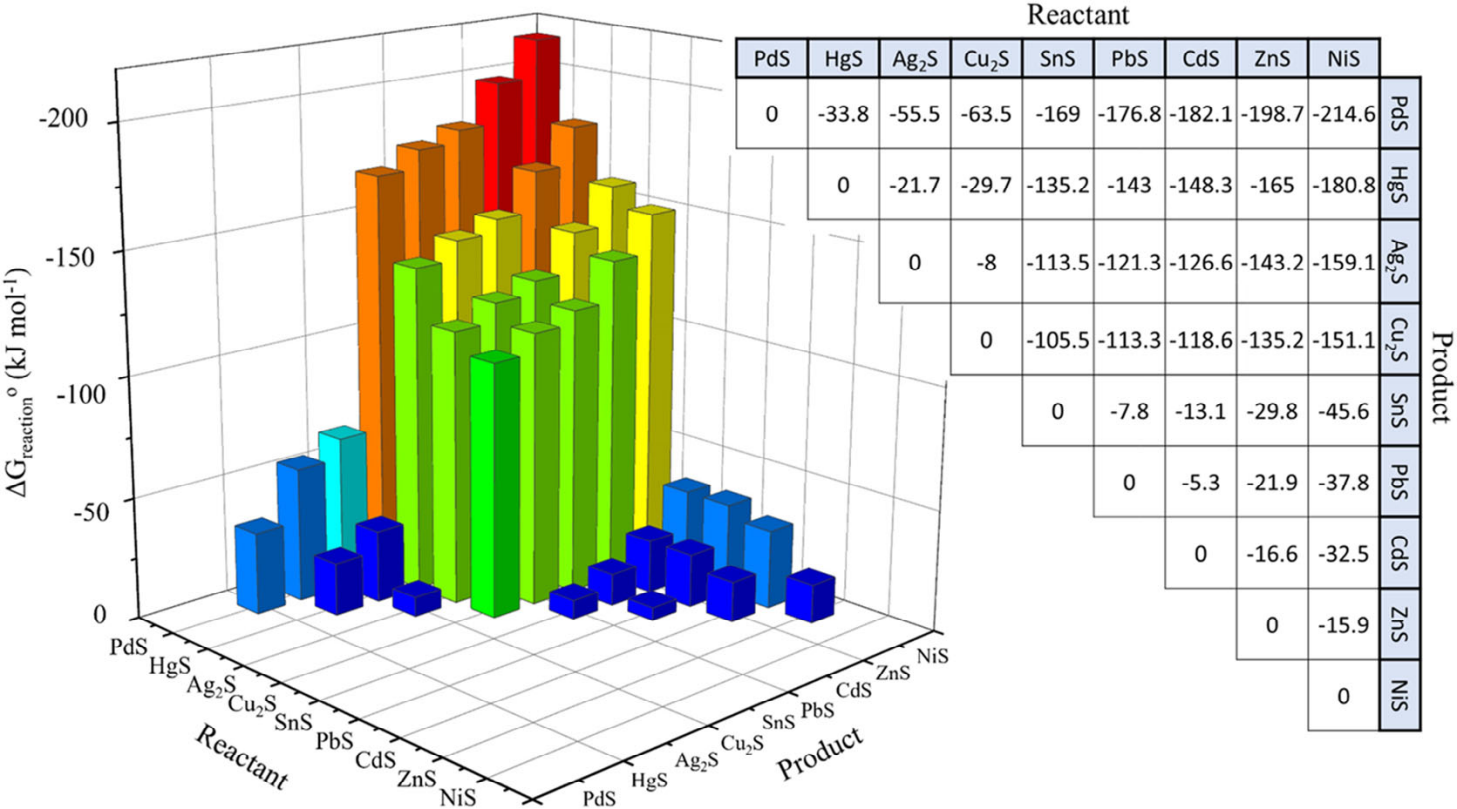
Calculated aqueous reaction Gibbs free energies, Δ*G*_reaction_^0^ (kJ mol^−1^), for the cation exchange of various metal sulfides. Reproduced with permission.^[^ ^12^ ^]^ Copyright 2019, American Chemical Society.

Based on the theories mentioned above, the simple aqueous ion‐exchange thermodynamics has been conveniently considered in selected types of metal sulfide phases (**Figure** **2**).^[^ ^12^, ^20^ ^]^ However, it must be noted that the trend of the calculations takes into account only the bulk lattice formation energies for each phase and aqueous redox potentials for the attendant metal ions. The practical CER in more complex conditions may significantly differ from the estimates. Therefore, in‐depth understanding is required to describe the actual reactions with other thermodynamic factors, such as bond dissociation energies (BDEs) linked to lattice energy, solvation energy related to solubility product constants (*K*_sp_), and Pearson's hard and soft acid and base theory (HSAB). A complete understanding of various factors makes thermodynamics more powerful for quantifying the variations in ion‐exchange reactions, offering basic guidelines for selecting suitable ligands and solvents.

The thermodynamic feasibility of cation exchange can be tentatively rationalized by the trend in solubility product constants (*K*_SP_) of the materials involved. It is known that an ionic solid with a relatively high *K*_sp_ can be spontaneously transformed through cation exchange to another ionic solid with a lower *K*_sp_ (see **Table** **1**). As reported, Ag_2_Se and HgSe phase with lower *K*_sp_ (3 × 10^−54^ and 4 × 10^−59^, respectively) was spontaneously formed by cation exchange from Cu_2_S (*K*_sp_: 2.5 × 10^−48^).^[^ ^21^ ^]^ In addition, the lattice energy (Δ*H*_latt_) of an ionic crystal is defined as the energy required to break the crystal apart into isolated ions at absolute zero temperature: A–X → A + X. This energy can give a measure of the strength of the chemical bonding among ions constituting the ionic solid, and thus, the higher lattice energy implies more stable crystal. Copper chalcogenides normally have lower Δ*H*_latt_ (Cu–Se, Cu–S) compared with various metal chalcogenides (M–Se, M–S) (see **Table** **2**). The energetic favors resulted in numerous metal sulfides synthesized by cation exchange using Cu_2_S templates.^[^ ^22^, ^23^, ^24^ ^]^ Similarly, estimating the BDEs is an alternative qualitative approach to determine the relative stabilities of reactants and products and thus evaluate the thermodynamics of the CER.^[^ ^25^ ^]^ Using tabulated values of BDE, the predicted relative stabilities of many compounds are in good agreement with the trend in Δ*H*_latt_. Unfortunately, the BDEs do not consider the crystal structures of exposed facets,^[^ ^21^ ^]^ which makes it still notoriously challenging to precisely evaluate the thermodynamic factors and predict the resulting NCs accurately and statistically.

**Table 1:** *K*_sp_ values of typical metal sulfides and experimental absolute hardness (*η*) of some typical cations and ligands^[^ ^26^, ^27^, ^28^ ^]^

Metal sulfides	*K*_sp_	Acid	Hardness [*η*]	Base	Hardness [*η*]
Cu_2_S	2.5 × 10^−48^	Cu^+^	6.28	C_6_H_5_NH_2_	4.4
CuS	6.3 × 10^−36^	Cu^2+^	8.27	C_6_H_5_SH	3.6
CdS	8 × 10^−27^	Co^2+^	8.22	C_6_H_5_OH	4.8
CdSe	4 × 10^−35^	Ni^2+^	8.50	CH_3_CHO	5.7
Ag_2_S	6.3 × 10^−50^	Cd^2+^	10.29	(CH_3_)_3_ P	5.9
Ag_2_Se	3 × 10^−54^	Ag^+^	6.96	(CH_3_)_2_S	6
ZnS	1.6 × 10^−24^	Zn^2+^	10.88	CH_3_Cl	7.5
ZnSe	3.6 × 10^−26^	Au^3+^	8.4	H_2_O	9.5

**Table 2:** Lattice energies (Δ*H*_latt_) and BDEs of metal chalcogenides (M–Y)^[^ ^29^, ^30^, ^31^ ^]^

Metal sulfides	BDEs [kJ mol^−1^]	Δ*H*_latt_
Cu_2_S	274.5 ± 14.6	2865
Cu_2_Se	255.2 ± 14.6	2936
CdS	208.5 ± 20.9	3460
CdSe	127.6 ± 25.1	3310
Ag_2_S	216.7 ± 14.6	2677
Ag_2_Se	210.0 ± 14.6	2686
ZnS	224.8 ± 12.6	3674
ZnSe	170.7 ± 25.9	−

Up to date, various factors are being studied to accurately predict the result of CERs, and reliable data inferred from experimental results are being accumulated. Boosted by the accumulated knowledge, the regiospecificity‐controlled CER has recently been attempted. In the next section, we systematically categorize the recent progress of the regiospecificity‐controlled CERs and discuss the advances in synthetic strategies.

## 3 Synthetic Strategies for Regiospecificity Control of Cation Exchange

### 3.1 Thermodynamic Stabilization

**Figure 3:**
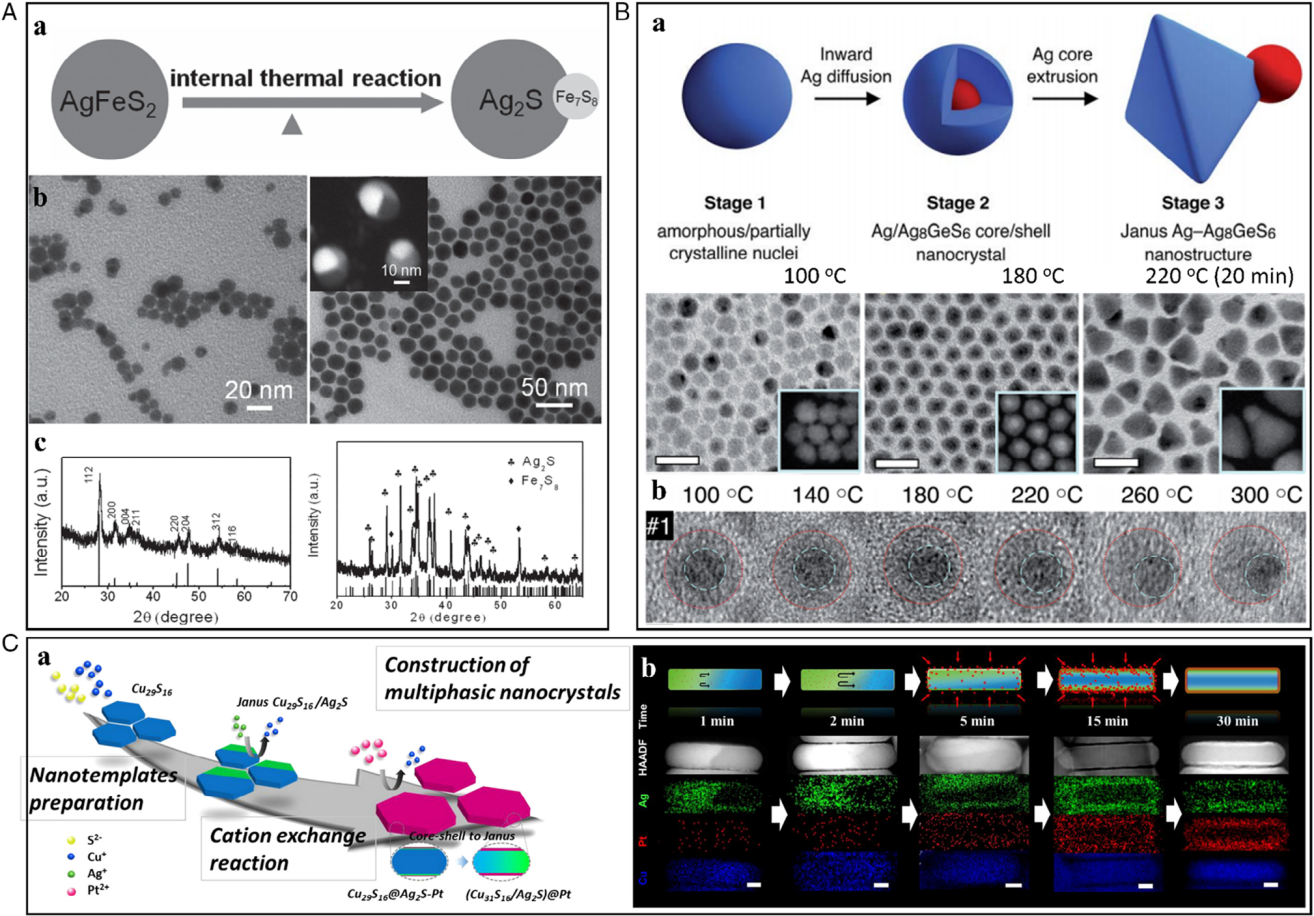
A) (a) Schematic illustration of the phase segregation process from AgFeS_2_ alloy NCs to Ag_2_S–Fe_7_S_8_ heterodimers. (b) Representative TEM images of AgFeS_2_ NCs and Ag_2_S–Fe_7_S_8_ heterodimers (inset: high‐angle annular dark‐field scanning transmission electron microscopy (HAADF‐STEM) image). (c) Corresponding powder X‐ray diffraction (PXRD) patterns of AgFeS_2_ NCs and Ag_2_S–Fe_7_S_8_ heterodimers. A) Reproduced with permission.^[^ ^32^ ^]^ Copyright 2013, Wiley‐VCH. B) (a) Schematic illustration with TEM images of the Ag diffusion and extrusion process from Ag_8_GeS_6_ NCs to Janus Ag/Ag_8_GeS_6_ heterostructures (Inset: HAADF‐STEM image). (b) HRTEM images of Ag/Ag_8_GeS_6_ core/shell structures heated to 300 °C within the electron microscope, where the apparent movement of the core to the outside of the particle is clearly visible with increasing temperature. B) Reproduced with permission.^[^ ^33^ ^]^ Copyright 2016, Royal Society of Chemistry. C) (a) Schematic illustration of the Janus‐to‐core@shell‐to‐Janus structural transformation of the Cu_2−*x*_S/Ag_2_S heterostructure by Pt. (b) HAADF‐STEM and energy‐dispersive X‐ray spectroscopy (EDS) elemental mapping images of the Janus to core@shell transformation of Cu_2−*x*_S/Ag_2_S to Cu_2−*x*_S@Ag_2_S–Pt. C) Reproduced with permission.^[^ ^23^ ^]^ Copyright 2019, American Chemical Society.

Nanomaterials, considerably affected by surface energy state due to the high surface area per volume, can take thermodynamically metastable phases, which is highly unlikely for bulk materials. Also, metastable NCs are facilely transformed to thermodynamically stable phases with sufficient energy inputs. The appearance of a metastable phase and the tendency to seek a thermodynamically stable phase in nanomaterials can be advantageously exploited to approach compositionally challenging hetero‐nanostructures. For example, metastable alloyed NCs can become stabilized by forming phasesegregated heterostructures, which would inevitably involve the migration of highly diffusive species within nanoparticles. The tendency to lower surface energy, minimization of lattice mismatch, phase stability at certain temperature and pressure, and atom miscibility among others are the main driving forces for NC phase transition. Han et al. reported a thermal‐reaction‐induced intraparticle segregation process from ternary AgFeS_2_ NCs to Ag_2_S–Fe_7_S_8_ heterodimers (**Figure** **3**A).^[^ ^32^ ^]^ In the ternary AgFeS_2_ NCs, Fe^3+^ ions with the smallest atomic size in the crystal structure could diffuse to the surface, driven by thermodynamic stabilization at elevated temperatures. The gradual component separation led to the formation of the Ag_2_S–Fe_7_S_8_ heterodimers eventually.

Embden et al. observed that thermal instability and Ostwald ripening could result in “self‐regulated” Ag elemental segregation processes from ternary Ag_8_GeS_6_ NCs to core/shell Ag/Ag_8_GeS_6_ and then Janus Ag/Ag_8_GeS_6_ hetero‐nanostructures (Figure 3B).^[^ ^33^ ^]^ The amorphous Ag–Ge–S NCs first nucleated and partially crystallized in the Ag_8_GeS_6_ composition and then gradually transformed to core/shell Ag/Ag_8_GeS_6_ hetero‐nanostructures by inward diffusion of Ag^+^ ions. Finally, intraparticle Ostwald ripening of the shell initiated on one side of the core, leading to extrusion of the Ag core to the surface, which resulted in complete transformation to Janus Ag/Ag_8_GeS_6_ heterostructures. Obviously, the thermally induced intraparticle segregation reduced the total free energy of the system by forming favorable heterointerfaces. The extrusion of the core to the outside of the particle was investigated by high‐resolution transmission electron microscopy (HRTEM) analysis through heating the sample to 300 °C within the electron microscope. Recently, our group reported an intraparticle segregation process from Janus Cu_29_S_16_/Ag_2_S to core/shell Cu_31_S_16_/Ag_2_S–Pt to Janus Cu_31_S_16_/Ag_2_S–Pt nanoplates and finally to Janus Cu_31_S_16_/Ag_2_S–Pt nanoplates (Figure 3C).^[^ ^23^ ^]^ The Janus Cu_29_S_16_/Ag_2_S nanoplates were obtained by partial cation exchange from Cu_29_S_16_ nanoplates. The Janus configuration was then transformed into a core/shell Cu_31_S_16_@Ag_2_S–Pt nanoplate by incorporating an epitaxial Pt overlayer. With the higher compatibility between Pt and Ag_2_S, an unprecedented cation migration of Cu^+^ ion and Ag^+^ ion in the sulfide matrix occurred and formed core/shell Cu_31_S_16_/Ag_2_S–Pt to release lattice strain between Ag_2_S and Cu_2_S in Janus configuration. However, when the surface Pt concentration was decreased due to the inward Pt diffusion, a drastic retransformation occurred from core/shell back to Janus as the phase‐segregated Janus structure was thermodynamically more favorable in the Cu–Ag system.^[^ ^34^, ^35^ ^]^

### 3.2 Kinetics Control

Many experimental parameters (such as types of templates, precursors, solvents, and additives) can affect the chemical equilibrium of colloidal ion‐exchange reactions considerably.^[^ ^36^ ^]^ Therefore, CERs often occur in a nonequilibrium state dominated by kinetics rather than thermodynamics. Ever since the first tunable and scalable synthesis of complex heterostructured NCs was reported,^[^ ^7^, ^11^ ^]^ the scope of accessible NCs has been dramatically expanded due to the newly gained insight on the cation diffusion process.^[^ ^6^ ^]^ More specifically, the cation diffusion kinetics is critically dependent on the vacancy density of the templates, because vacant cation sites create cation diffusion pathways.^[^ ^37^ ^]^ The high density of vacancies can lower the kinetic barrier of cation exchange, thereby boosting the ion diffusion rate.^[^ ^24^ ^]^ Hence, the investigations on the influence of kinetics factors, including the characteristics of templates, are equally important as thermodynamic aspects in predicting the direction of colloidal ion‐exchange reactions. This section discusses the factors related to the kinetics for achieving CERs and strategies for controlling the regiospecificity.

#### 3.2.1 Template Shape

Crystal structure retention is a vital feature of cation exchanges, where the persistence of the continuous anion sublattice allows the regiospecificity control by forming interfaces in specific crystallographic directions.^[^ ^38^ ^]^ Therefore, the prudent choice of template plays a significant part in the successful regiospecificity‐controlled CERs.

**Figure 4:**
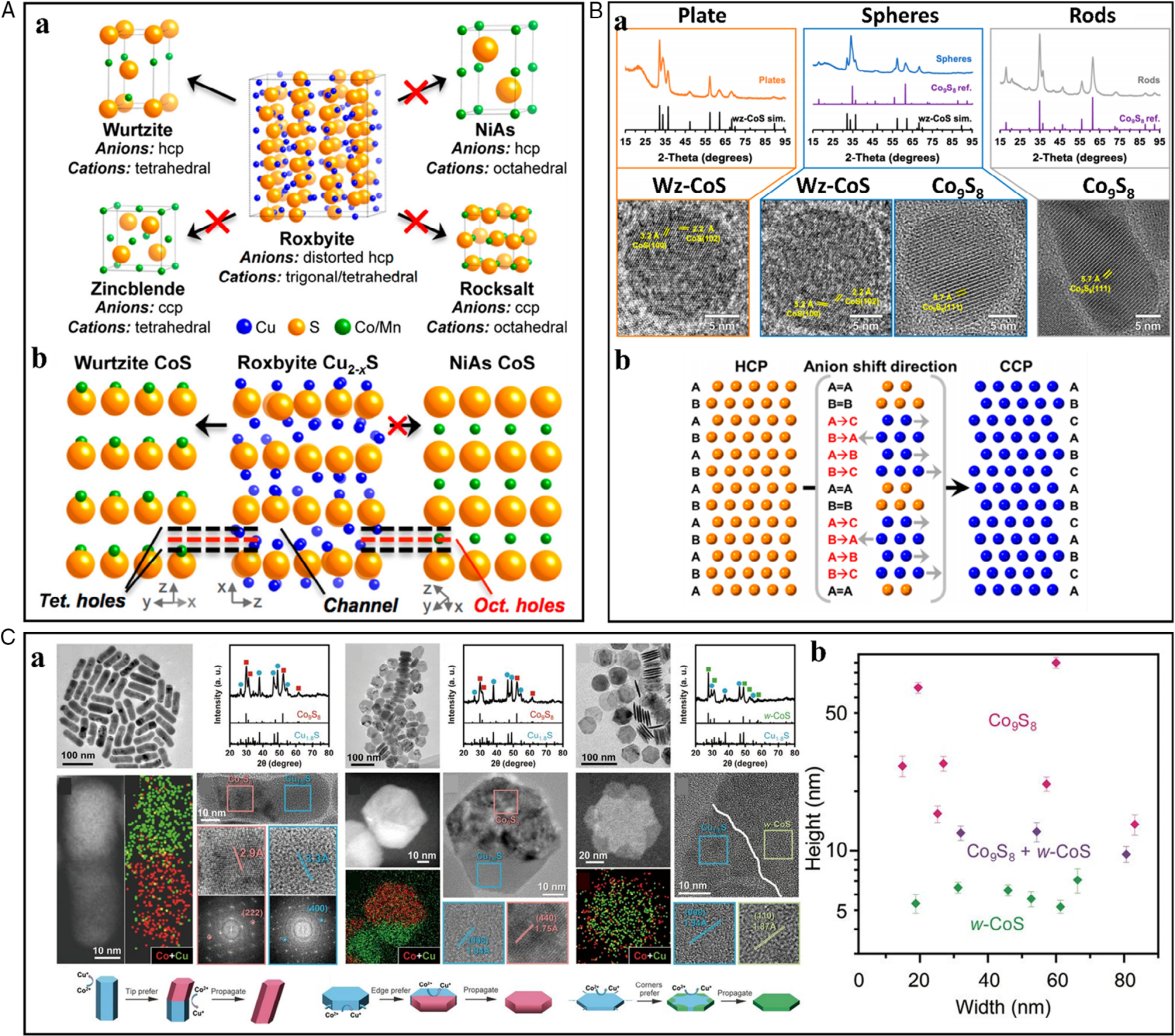
A) (a) Selective phase transformation of roxbyite Cu_2−*x*_S into wurtzite structure relative to the zincblende, and rocksalt polymorphs. (b) Crystallographically related projections of the wurtzite CoS, roxbyite Cu_2−*x*_S, and NiAs CoS structures, emphasizing the vertical registries of the tetrahedral and octahedral holes (black and red dashed lines, respectively), as well as the cation vacancies in roxbyite that create open channels. A) Reproduced with permission.^[^ ^42^ ^]^ Copyright 2016, American Chemical Society. B) (a) PXRD and TEM analysis of resulting cobalt sulfides transformed from Cu_1.8_S in plate, sphere, and rods shapes. b) Crystal illustration shows the shift of the anion frameworks. B) Reproduced with permission.^[^ ^43^ ^]^ Copyright 2021, American Chemical Society. C) (a) HRTEM and PXRD analysis of morphology‐dependent phase selectivity of CER from Cu_1.8_S to cobalt sulfides. (b) Height–width plots of host Cu_1.8_S NCs, and corresponding cobalt sulfide phases after the CERs. C) Reproduced with permission.^[^ ^44^ ^]^ Copyright 2021, The Authors, published by AAAS.

In general, metastable nanotemplates exhibit the potential to undergo diverse transformative and phasic transitions. Among those, copper sulfides (Cu_2−*x*_S) NCs have emerged as an ideal template for producing numerous new materials due to their intrinsic metastability, defective crystal features, and high mobility of Cu ions, enabling efficient interstitial diffusion and substitution of incoming cations.^[^ ^5^, ^8^, ^39^ ^]^ Therefore, many phases, such as covellite (CuS), roxbyite (Cu_1.81_S), and chalcocite (Cu_2−*x*_S), and structured Cu_2−*x*_S NCs have been used to produce derivative NCs having unique compositions and sophisticated heterostructures.^[^ ^39^, ^40^ ^]^ The different phases and structures of Cu_2−*x*_S have distinctively different exposed crystal facets, surface energy, and reactivity. The roxbyite system has been intensively investigated with its anion sublattice consisting of a distorted hexagonal‐close‐packed (HCP) structure and the cation lattice consisting of mixed trigonal and tetrahedral structures.^[^ ^41^ ^]^ Due to the analogous anion frameworks between wurtzite and roxbyite, the wurtzite crystal structure is generally preferred for the cation‐exchanged products from roxbyite even though the phase is metastable in bulk (**Figure** **4**A).^[^ ^42^ ^]^ Interestingly, it has recently been revealed that the resultant phases after CER could be influenced by the template morphologies. Butterfield et al. observed unconventional anion sublattice reconstruction from HCP (roxbyite Cu_1.8_S) to cubic‐close‐packed (CCP, Co_9_S_8_) rather than wurtzite (HCP, CoS), which strongly depended on the height of the host roxbyite Cu_1.8_S.^[^ ^43^ ^]^ They investigated the morphology‐dependent phase selectivity for wurtzite CoS (HCP) and pentlandite Co_9_S_8_ (CCP) through the Co^2+^ exchange on roxbyite Cu_1.8_S plates, spheres, and rods. The plates formed wurtzite CoS, the rods formed Co_9_S_8_, and the spheres formed both wurtzite CoS and Co_9_S_8_. The plates, spheres, and rods had nearly identical widths but increased in length in the direction of the close‐packed planes stacks, influencing the anions shifting from HCP to CCP during cation exchange (Figure 4B). This morphology‐dependent behavior, correlating with the number of stacked close‐packed planes, relied on an anion sublattice rearrangement that was concomitant with cation exchange. In the same year, Li et al. also reported a similar phenomenon of the effect of template shape on the resultant phase during cation exchange from roxbyite Cu_1.8_S with Co^2+^.^[^ ^44^ ^]^ The experiments and theoretical calculations revealed that thermodynamically unstable wurtzite CoS could be stabilized by the robust nature of S^2−^, the anion framework of plate morphology. However, in the case of rods with large heights, the S^2−^ anion framework could be easily reconstructed due to the lower kinetic barrier for S^2−^ reconstruction, resulting in transformation into thermodynamically preferred Co_9_S_8_, not the wurtzite CoS (Figure 4C). Further investigation of Mn^2+^, Zn^2+^, and Ni^2+^ cation exchanges revealed that the resultant crystal structures could also be modulated by other factors such as lattice volume, thermodynamic stability, and coordination environment. This template morphology‐dependent crystal phase of CER product scan might open a broader opportunity for the synthetic strategies of regiospecificity‐controlled NCs.

#### 3.2.2 Crystal Structures

**Figure 5:**
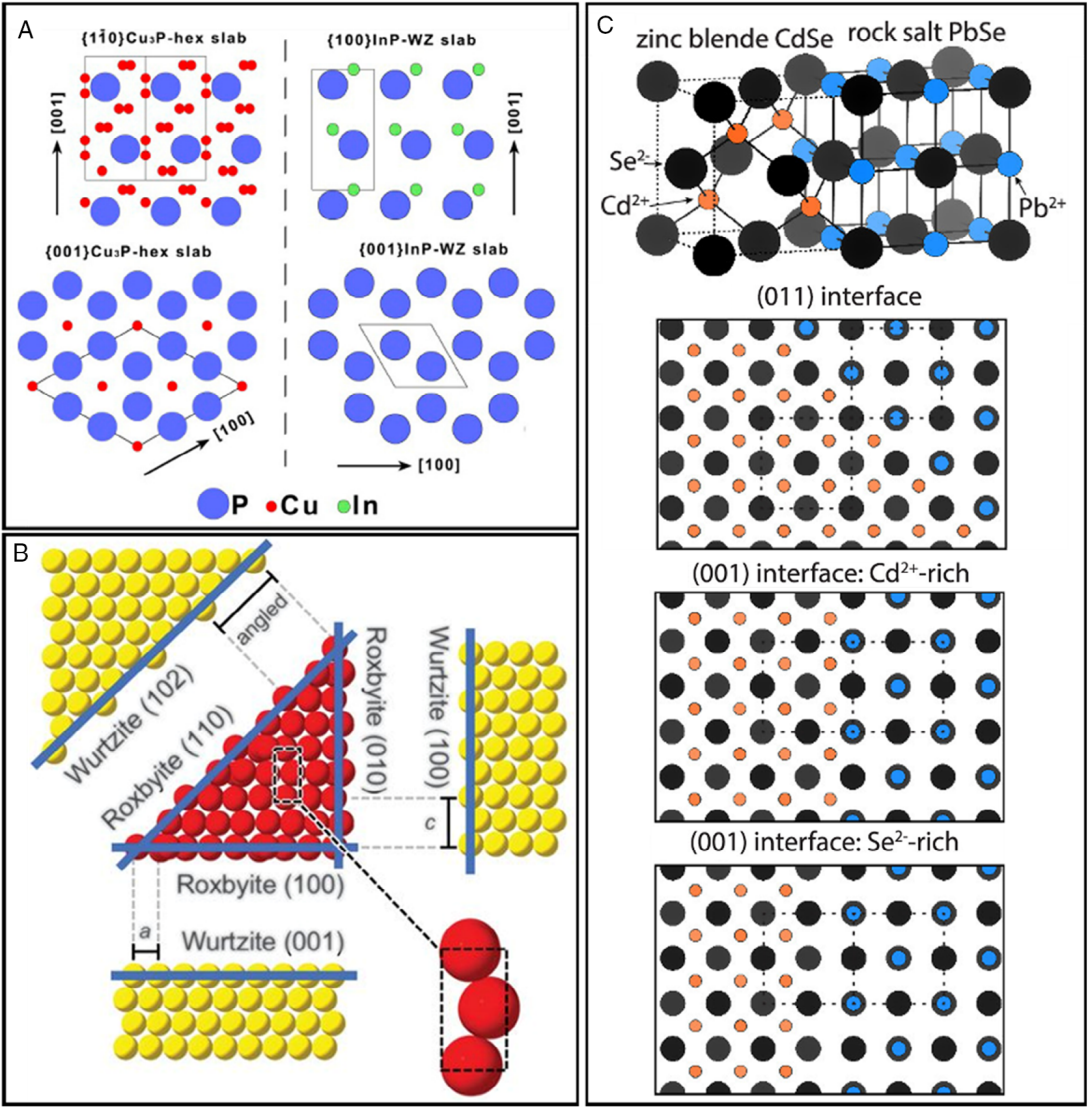
A) Atomic crystal schematics of [1$\bar{1}0$] and [001] lattices of hexagonal Cu_3−*x*_P and [100], [001] lattices of wurtzite InP. Reproduced with permission.^[^ ^14^ ^]^ Copyright 2015, American Chemical Society. B) Projections of the interface between the crystal planes of wurtzite ZnS and roxbyite Cu_1.8_S. Reproduced with permission.^[^ ^7^ ^]^ Copyright 2020, The Authors, published by AAAS. C) Schematic depictions of heterostructures from zinc blende–CdSe and rock salt–PbSe with a continuous anion (selenium) sublattice (black spheres). Reproduced with permission.^[^ ^16^ ^]^ Copyright 2022, American Chemical Society.

Understanding the crystal lattice structure is very important for controlling ion diffusion rate and regiospecificity. Different template morphologies have their own respective exposed crystal planes, and the interface with the resulting phase is usually formed along the lattice direction with the smallest lattice mismatches.^[^ ^7^, ^14^ ^]^ In the case of Cu_3−*x*_P hexagonal plates as templates, the CER with In^3+^ initiated from the corners of the NCs and gradually propagated toward the center. Trizio et al. investigated the epitaxial relationship between the two phases. They revealed that the interface (100) InP || (2$\bar{1}0$) Cu_3−*x*_P and (001) InP || (001) Cu_3−*x*_P forms because the anions sublattices are preserved from the directions (**Figure** **5**A).^[^ ^14^ ^]^ Later, Koh et al. reported a more detailed CER mechanism of Cu_3−*x*_P and In^3+^.^[^ ^45^ ^]^ In partial CER, the direction of the interface formed was the same as before. However, in the case of a complete CER, cracked and hollow shapes were observed due to the formation of Cu^+^ vacancies, followed by the vacancy coalescence.

**Figure 6:**
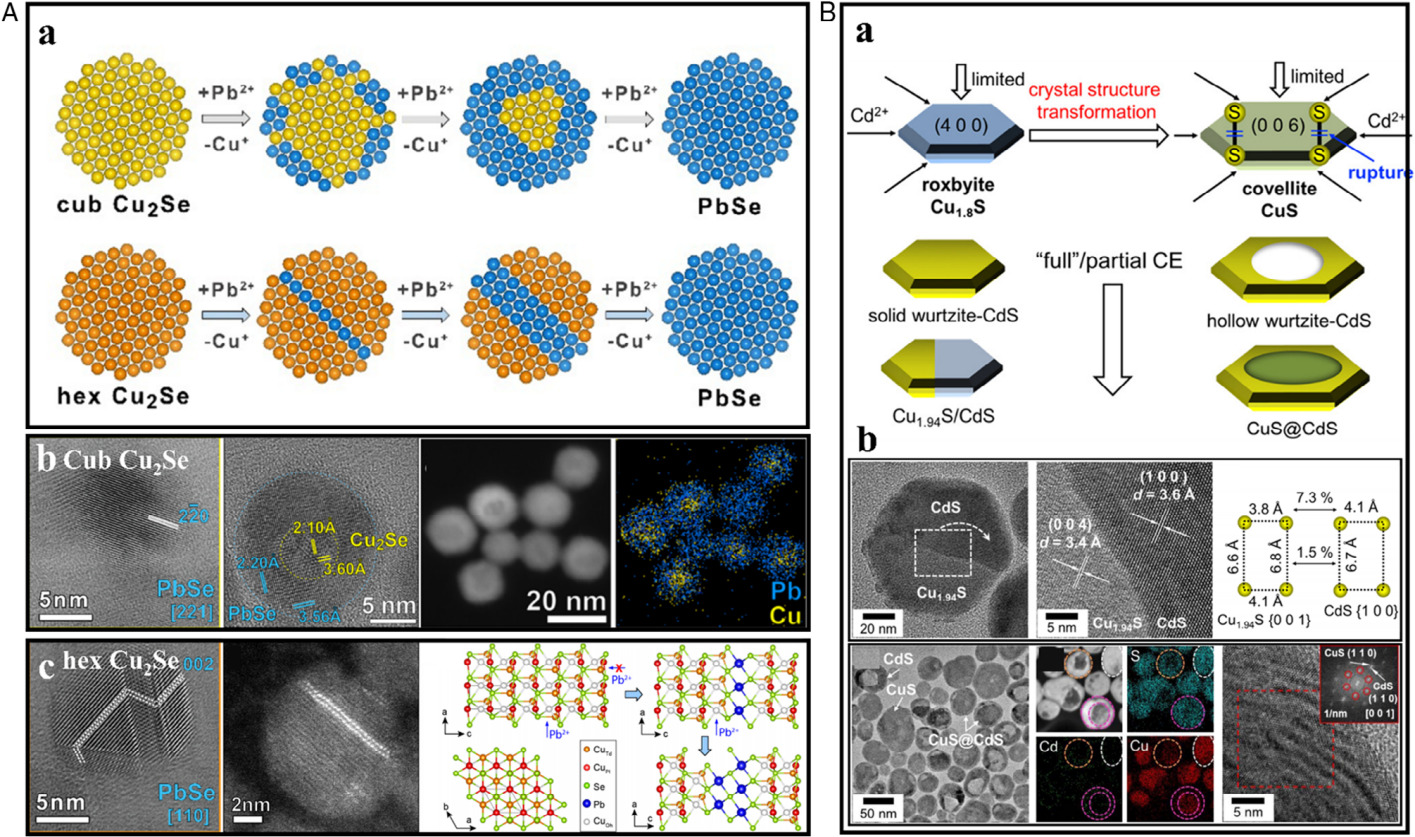
A) (a) Schematic illustration of cation exchange between either cubic or hexagonal Cu_2_Se NCs and Pb^2+^ cations. (b) HRTEM analysis, HAADF‐STEM image, and EDS elemental mapping images of PbSe NCs obtained starting from cubic Cu_2_Se. (c) HRTEM and HAADF‐STEM images; atomic schemes representing the hexagonal Cu_2_Se structure. A) Reproduced with permission.^[^ ^48^ ^]^ Copyright 2017, American Chemical Society. B) (a) Schematic representation of the full/partial Cd^2+^‐exchange reaction using roxbyite Cu_1.8_S and covellite CuS nanodisks. (b) Corresponsindg HRTEM analysis of Janus Cu_1.94_S/CdS and core@shell CuS@CdS NCs. B) Reproduced with permission.^[^ ^49^ ^]^ Copyright 2022, American Chemical Society.

During a partial CER, the interface is formed along the ion‐diffusion direction, which tends to create a continuous anion sublattice to reduce the lattice mismatch. For example, the Zn^2+^ exchange on roxbyite Cu_1.8_S nanorod created slanted interfaces between Cu_1.81_S (110) and wurtzite ZnS (102) due to their analogous anion sublattices (Figure 5B). Zinc blende CdSe and rock salt PbSe had three similar anion sublattices of (001), (011), and (001), which led to the formation of more complex heterostructures (Figure 5C).^[^ ^16^ ^]^ This CER principle has been expanded and studied as a methodology to control regiospecificity.^[^ ^46^, ^47^ ^]^ The influence of different crystal structures of the reacting platform on the diffusive abilities of cations was reported by Gariano et al. in the case of cation exchange between Cu_2_Se NCs and Pb^2+^.^[^ ^48^ ^]^ As shown in **Figure** **6**A, upon exposure to Pb^2+^ cations, the cubic Cu_2_Se host NCs resulted in the formation of Cu_2_Se/PbSe core/shell heterostructures due to the low diffusivity of Pb^2+^ ions inside the cubic Cu_2_Se NCs. However, in the case of hexagonal Cu_2_Se NCs, the entrance of Pb^2+^ ions generated PbSe stripes “sandwiched” between hexagonal Cu_2_Se domains where Pb^2+^ ions preferentially diffused through specific (a, b) planes of the hexagonal Cu_2_Se structure. As mentioned earlier, copper sulfide can have several phases depending on the ratio of Cu to S with different crystal structural characteristics. Chen et al. investigated the Cd^+^‐exchange reaction on covellite CuS and roxbyite Cu_1.8_S nanotemplate.^[^ ^49^ ^]^ While Janus and solid wurtzite CdS nanodisks were formed by partial and full Cd^2+^ exchange on roxbyite Cu_1.8_S, respectively, the hollow wurtzite CdS and core/shell CuS/CdS were obtained through different cation‐exchange mechanism (Figure 6B). They revealed that the disulfide (S–S) bonds in CuS dramatically affected the cation exchange dynamics and pathways. The sluggish S–S splitting reduced the cation exchange kinetics, resulting in the delay of the exchange rate between Cu^2+^ and Cd^2+^ along the lateral directions and the formation of core/shell structure with stepwise rupture of disulfide bonds (Figure 6C).

**Figure 7:**
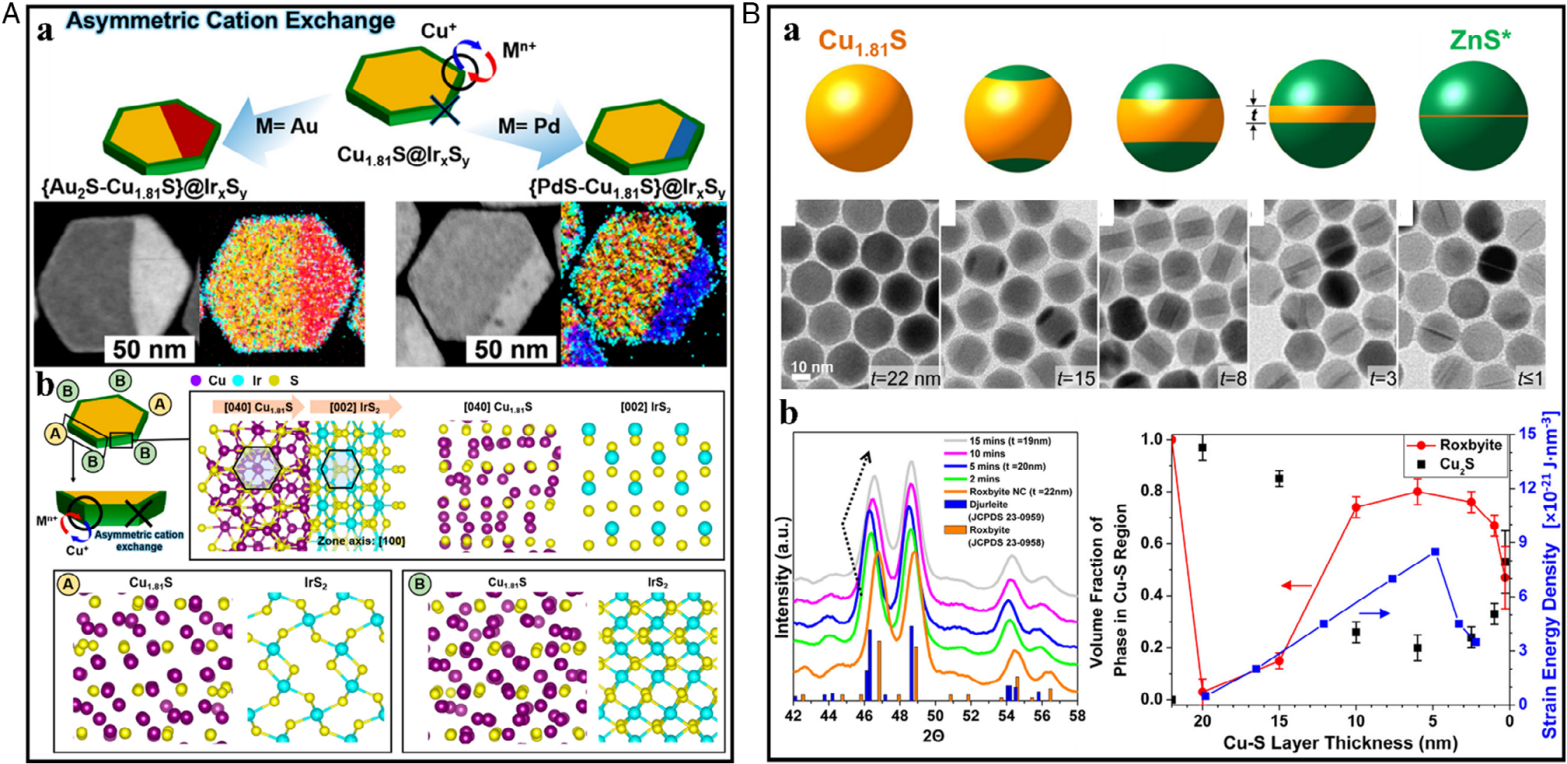
A) (a) Schematic illustration, HAADF‐STEM, and   EDS   elemental mapping image of Au and Pd cation‐exchange Cu_1.81_S@Ir_*x*_S_*y*_. (b) Atomic orientations of Cu_1.81_S@Ir_*x*_S_*y*_ in different corners showing different vacant density. A) Reproduced with permission.^[^ ^50^ ^]^ Copyright 2018, American Chemical Society. B) (a) Cation exchange transformation of copper sulfide NCs into dual‐interface heterostructured particles with zinc sulfide caps. (b) PXRD analysis and strain energy density showing the phase fraction in the copper sulfide. B) Reproduced with permission.^[^ ^15^ ^]^ Copyright 2014, American Chemical Society.

Park et al. developed a synthetic strategy to provide regiospecific cation exchange by forming core–crown Cu_1.81_S/Ir_*x*_S_*y*_ structures, imposing a significant kinetic hurdle to specific sites, forming unique heterostructures of (Au_2_S–Cu_1,81_S)@Ir_*x*_S_*y*_ and (PdS–Cu_1,81_S)@Ir_*x*_S_*y*_, respectively (**Figure** **7**A).^[^ ^50^ ^]^ Compared with Cu_1.81_S with an identical crystal structure at each corner, Ir_*x*_S_*y*_ has different vacant densities at the corner, which could induce low‐energy barriers for cation diffusions. As a result, the additional Ir_*x*_S_*y*_ crown on Cu_1.81_S played the role of ion filter, providing regiospecificity‐controlled CERs, while hexagonal Cu_1.81_S without Ir_*x*_S_*y*_ crown showed isotropic Au exchange from every corner.^[^ ^51^ ^]^

A strain between different lattice structures can also be a key factor leading to new regiospecificity‐controlled designs. The sandwiched structure appeared when Cu_1.81_S was exchanged with Zn^2+^ due to the similar structure of Cu_1.81_S and resulting wurtzite ZnS.^[^ ^15^ ^]^ As the CER initiated, the roxbyite (Cu_1.81_S) phase of copper sulfide showed a complete phase transition to djurleite (Cu_1.94_S)/low chalcocite (Cu_2_S), a thermodynamically more stable phase than roxbyite. The djurleite (Cu_1.94_S)/low chalcocite (Cu_2_S) underwent a second phase transformation back to roxbyite to minimize this strain energy, which shared a similar sulfur sublattice to wurtzite ZnS (Figure 7B).^[^ ^15^ ^]^ This study indicates that the lattice mismatch‐dependent phase selectivity can be a facile strategy to reach desired phases.

#### 3.2.3 Vacancies and Regiospecificity

**Figure 8:**
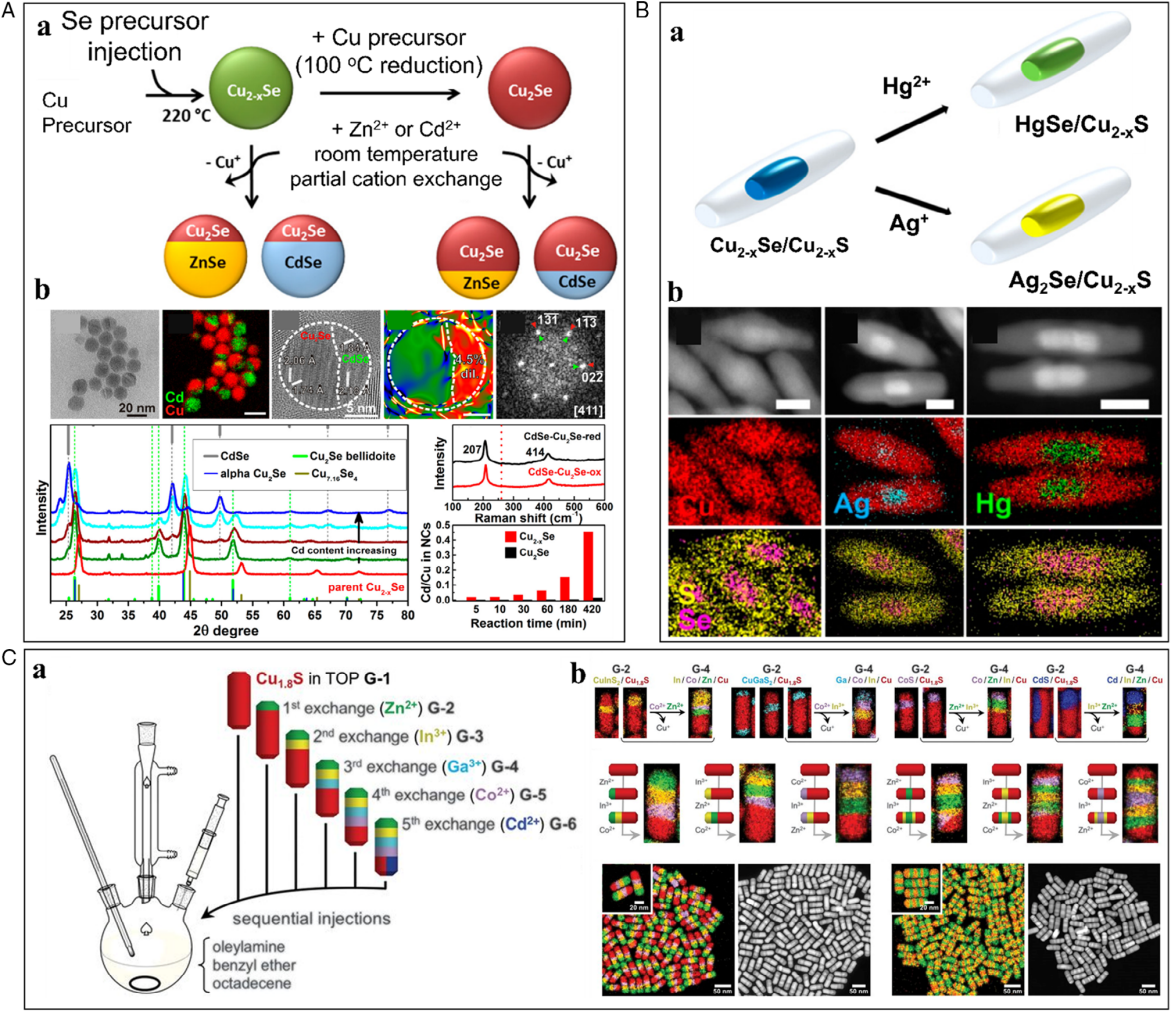
A) (a) Schematic illustration of selective cation exchange using defective Cu_2−*x*_Se and Cu_2_Se, respectively. (b) HRTEM, PXRD analysis of Zn^2+^, Cd^2+^‐exchanged Cu_2−*x*_Se, and Cu_2_Se NCs. A) Reproduced with permission.^[^ ^24^ ^]^ Copyright 2015, American Chemical Society. B) (a) Schematic illustration of selective cation exchange in the core region of Cu_2−*x*_Se/ Cu_2−*x*_S core/shell NCs. (b) STEM–EDS element maps of Hg^2+^‐ and Ag^+^‐exchanged HgSe/Cu_2−*x*_S and Ag_2_Se/Cu_2−*x*_S, respectively. B) Reproduced with permission.^[^ ^21^ ^]^ Copyright 2015, American Chemical Society. C) (a) The reaction setup for sequential CERs to transform Cu_1.8_S into ZnS–CuInS_2_–CuGaS_2_–CoS–(CdS–Cu_1.8_S). (b) STEM–EDS element maps of six distinct nanorods containing various spatial arrangements of ZnS, CuInS_2_, CoS, and Cu_1.8_S. C) Reproduced with permission.^[^ ^7^ ^]^ Copyright 2020, The Authors, published by AAAS.

The presence of a large density of cation vacancies significantly accelerates ion diffusion by lowering the activation energy along the defective pathway. Thus, the different densities of vacancies can considerably differentiate the ion‐exchange kinetics, achieving selective ion diffusion. The effect of vacancies during cation exchange was first verified by Lesnyak et al. Under identical reaction conditions, Zn^2+^ and Cd^2+^ have been exchanged on Cu_2_Se and Cu_2−*x*_Se with a higher vacancy density.^[^ ^24^ ^]^ Indeed, the exchange was faster and could readily reach completion when performed on a highly vacant template (Cu_2−*x*_Se), which indicated that vacancy diffusion was one of the main drivers of cation exchange (**Figure** **8**A). Furthermore, the phenomenon also appeared in another work of heterostructures of Cu_2−*x*_Se/Cu_2−*x*_S templates.^[^ ^21^ ^]^ Miszta et al. reported selective cation exchange of Ag^+^ and Hg^2+^ in the core region of Cu_2−*x*_Se/Cu_2−*x*_S core/shell NCs. The larger copper density of Cu_2−*x*_Se in the core resulted in the focused diffusion of guest cations into the core (Figure 8B). In this regard, the preferential CER on the more defective templates allows the regiospecficifity‐controlled multiple cation exchanges by sequential exchange strategies. For example, Steimle et al. reported the rational construction of 65 520 distinct multicomponent metal sulfide nanorods by applying up to seven sequential CERs using Cu_1.8_S nanorods (Figure 8C).^[^ ^7^ ^]^ That was achieved because partial cation exchanges occur preferentially in the remaining Cu_1.8_S region with a high vacancy density. The internal interfaces have been regiospecifically formed according to the compatibility of crystal structures between the resultant metal sulfide phases. Thus, it is evident that the regiospecificity in the cation exchange can be modulated by the heterostructured templates having a different degree of vacancy density.

## 4 Applications of Heterostructured NCs

With fine control over the shape, size, and composition, the colloidal NCs pose attractive properties suitable for applications like photoluminescence (PL), photocatalysis, optoelectronics, sensors, and electrochemical energy conversion and storage.^[^ ^9^, ^52^ ^]^ As discussed in the previous sections, ion‐exchange reactions are an efficient and cost‐effective post‐synthetic method to obtain precise control over the characteristics of the NCs. Furthermore, by regiospecific control of the ion‐exchange strategies, their properties could be fine tuned for a particular type of application. This section highlights the potential applications of the regiospecifically designed, complex heterostructures such as core–shell, striped, segmented, sandwich, and hollow NCs.

### 4.1 Photoluminescence

**Figure 9:**
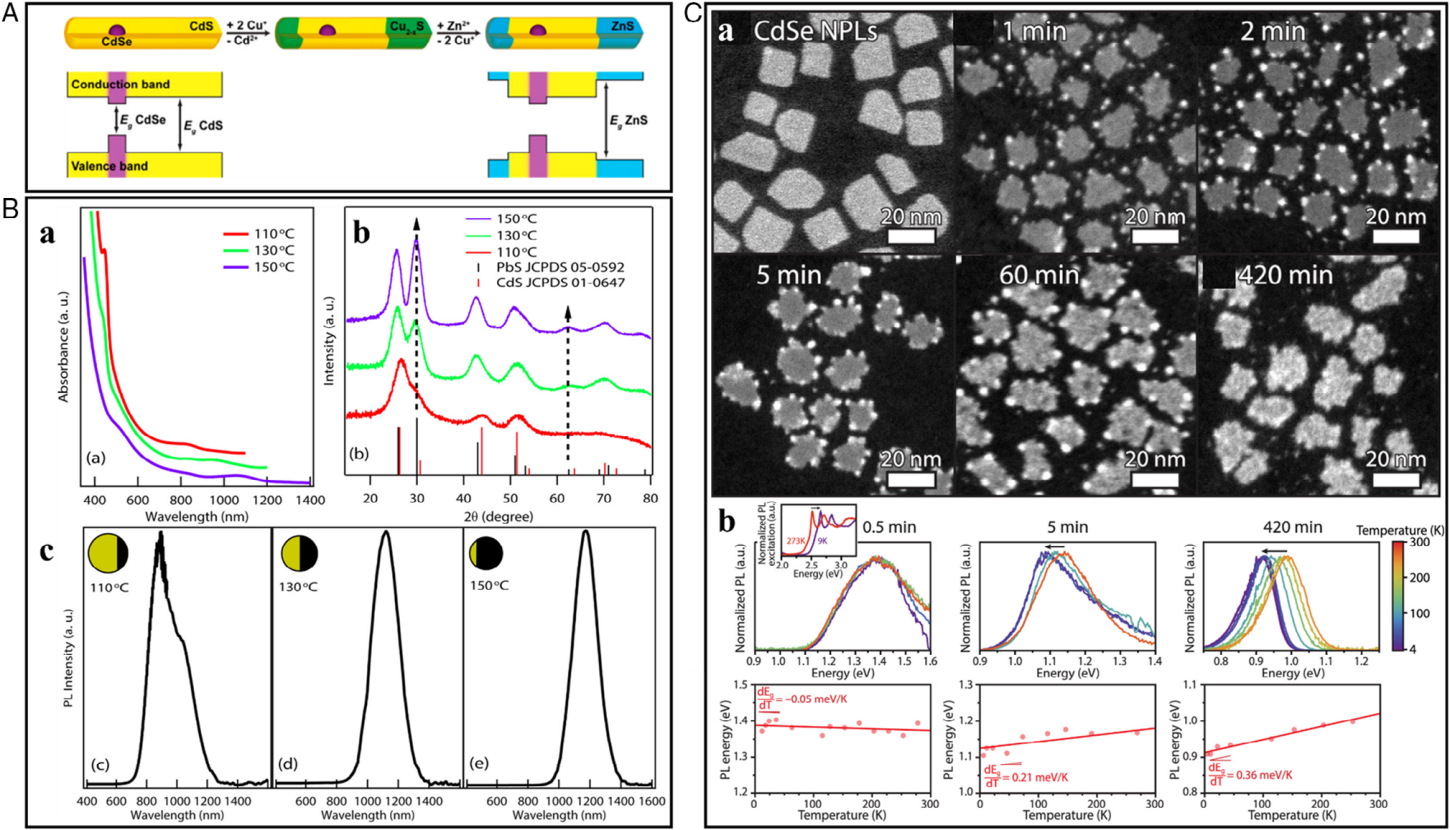
A) Schematic representing the tailoring of bandgap with ion‐exchange reactions. Reproduced with permission.^[^ ^53^ ^]^ Copyright 2014, American Chemical Society. B) (a) Absorption spectra, (b) PXRD patterns, and c) PL spectra of the CdS/PbS heterostructures synthesized at different temperatures. The ratio of PbS:CdS increases with higher temperatures, which is schematically shown by the spheres (yellow represents CdS and black represents PbS). B) Reproduced with permission.^[^ ^54^ ^]^ Copyright 2015, American Chemical Society. C) (a) HAADF‐STEM images and (b) PL emission spectra of 2D CdSe–PbSe‐heterostructured NCs after Pb^2+^/Cd^2+^ CER, and corresponding peak position versus the temperature. C) Reproduced with permission.^[^ ^16^ ^]^ Copyright 2022, American Chemical Society.

The luminescence of the inorganic NCs can be altered by modifying their composition and morphology. CdSe@CdS/ZnS hetero‐nanorods realized through the regioselective sequential CER having a segmented structure with a CdSe core embedded in a CdS rod terminated epitaxially with ZnS, exhibiting high PL and quantum efficiencies.^[^ ^53^ ^]^ The ZnS at the ends of the rod is a higher‐bandgap semiconductor than the CdS and thus confines the charge carriers at the CdSe core (**Figure** **9**A). This configuration provided good electronic insulation to the CdSe core, which dominated the emission behavior of the overall structure to result in the high‐efficiency absorption and emission spectra of the core. Zhang et al. reported that the CdS/PbS and CdSe/PbSe Janus‐type heterostructures exhibit a nontrivial PL behavior with the increase in the Pb:Cd ratio.^[^ ^54^ ^]^ They reported the anisotropic exchange of Cd^2+^ to Pb^2+^ along the <111> direction with a sharp epitaxial interface at (111). In the PL emission spectra of CdS/PbS heterostructures, the band‐edge emission of the PbS redshifted with the increase in the PbS domain. Similarly, the CdSe band emission and its native trap emission in the PL emission of CdSe/PbSe heterostructure became negligible with the addition of PbSe (Figure 9B). More recently, Salzmann et al. reported the PL properties of CdSe–PbSe heterostructures. The regiospecific cation exchange of Pb^2+^ for Cd^2+^, starting at the vertical facets of CdSe nanoplatelets, was revealed through the crystallographic study of intermediate reaction products.^[^ ^16^ ^]^ The emission from the PbSe domains was indicated by the strong redshifted broad PL peak at 1.33 eV in the emission spectrum (Figure 9C). A dark–bright exciton‐state splitting was present in PbSe nanoplatelets with 3D quantum confinement. They also found that the 2D CdSe–PbSe heterostructures have optical properties with swift exciton energy transfer from the CdSe lattice to the PbSe domains, which were also temperature and time dependent. Cu_3−*x*_P NCs with localized surface plasmon resonance (LSPR) exhibited optical features in the near‐infrared (NIR) region.^[^ ^14^ ^]^ When the Cu_3−*x*_P NCs were subjected to CERs with In^3+^, they formed wurtzite InP hexagonal platelets, regioselectively starting at the corners of the NCs and gradually evolving toward the center. Like their parent structures, the wurtzite InP NCs did not show any feature in the NIR region. The InP hexagonal platelets had an absorption edge at 800 nm corresponding to a bandgap of 1.55 eV. However, this value was larger than the bandgap (832 nm and 1.49 eV) of bulk wurtzite InP, indicating the quantum confinement of carriers in the wurtzite InP hexagonal platelet NCs.

### 4.2 Photocatalysis

By hybridizing the semiconductors and constructing the heterostructures through CERs, high‐performance photocatalysts with improved charge separation efficiency and photoresponse time can be developed. However, constructing controlled interfaces with small lattice mismatches has been known as one of the most challenging points in this field.^[^ ^17^, ^55^ ^]^ The ion‐exchange reaction is an effective method to produce precisely controlled, high‐quality interfaces. Yuan et al. hypothesized that the tetrahedrally coordinated Mn^2+^ could be used to incorporate cations as they have a great potential to coexist with copper sulfide NCs to form Janus structures.^[^ ^56^ ^]^ The Janus‐like *γ*‐MnS–Cu_7_S_4_ nanostructures synthesized with high‐quality interfaces expose both the semiconductors that is roxbyite Cu_7_S_4_ and *γ*‐MnS on the surface for broadband photocatalytic hydrogen evolution. Liu et al. reported that the Au/CdSe Janus nanospheres produced through CERs had a photocatalytic activity 3.9 times higher than other hybrid structures like heterodimers, symmetric double headed, and multiheaded produced by manipulating the pH of the solution.^[^ ^57^ ^]^ This increase in the photocatalytic activity was attributed to the flat and high‐quality interface between Au and CdSe, contributing to improved interfacial electron transfer efficiency. A hollow CuInS_2_ nanododecahedron with uniform composition and morphology obtained through the Kirkendall effect and cation exchanges in Cu_2‐x_S nanododecahedrons also showed excellent photocatalytic properties.^[^ ^58^ ^]^ This increase in properties was attributed to the enhanced light harvesting and photogenerated charge carrier separation with the increase in the degree of hollowing.

**Figure 10:**
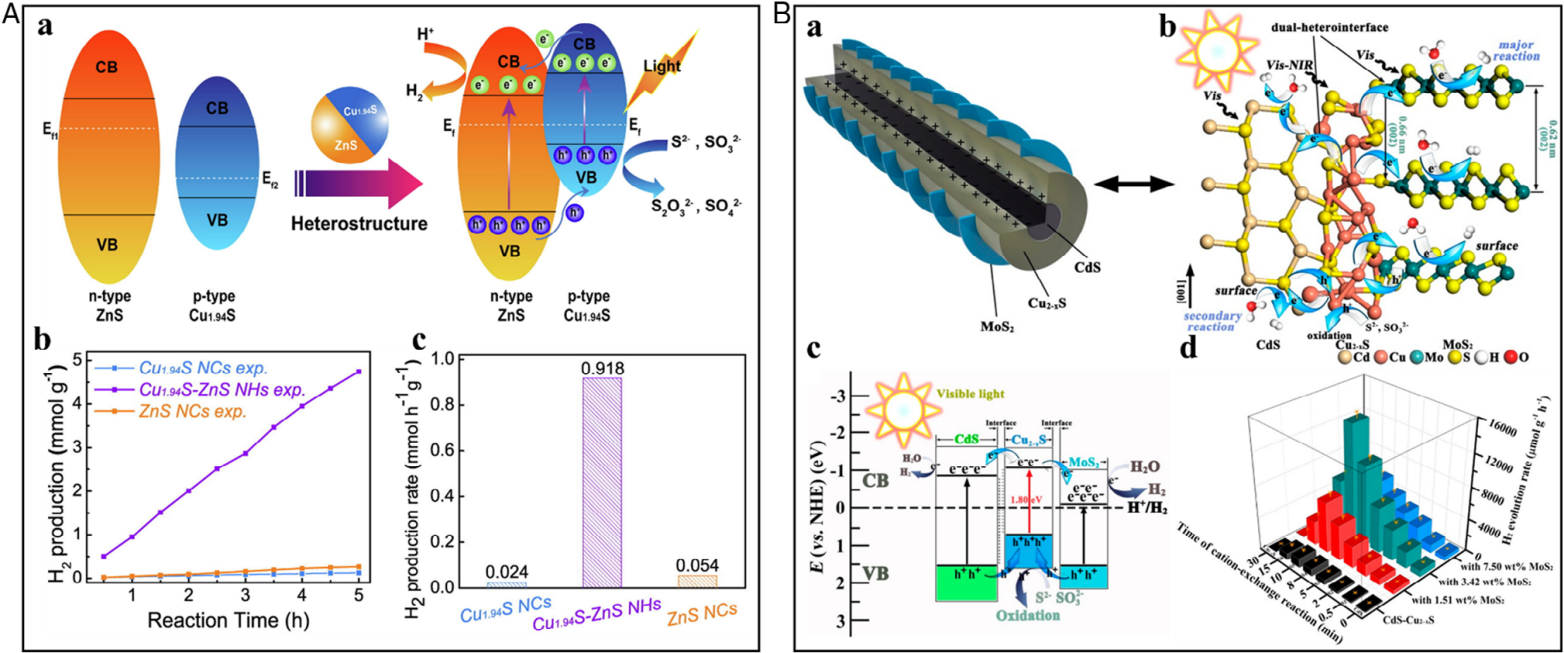
A) (a) Schematic representing the charge transfer mechanism in Cu_1.94_S–ZnS heterostructures for photocatalytic hydrogen production. (b) Photocatalytic hydrogen production under the simulated solar light and (c) the corresponding hydrogen production rate. A) Reproduced with permission.^[^ ^17^ ^]^ Copyright 2021, Elsevier Ltd. B) (a) Schematic illustration of stratified CdS–Cu_2−*x*_S/MoS_2_ NCs and (b,c) proposed mechanism of photocatalytic H_2_ production. (d) Photocatalytic H_2_ evolution activities of different catalysts under visible light irradiation (*λ* > 400 nm). B) Reproduced with permission.^[^ ^60^ ^]^ Copyright 2020, American Chemical Society.

Guo et al. reported the stoichiometrically limited CER to produce Janus Cu_1.94_S–ZnS heterostructures.^[^ ^17^ ^]^ The heterostructures showed improved photocurrent density in the presence of light, indicating their increased ability to harvest light and separate the photogenerated electron–hole pairs. Understanding the charge transfer mechanism from Cu_1.94_S to ZnS in the Cu_1.94_S–ZnS heterostructures was crucial in elucidating the improved hydrogen evolution activity. **Figure** **10**A(a) illustrates the donor–acceptor model depicting the formation of a p–n junction when Cu_1.94_S and ZnS are combined through partial cation exchange. To equilibrate the Fermi‐energy levels of the formed Cu_1.94_S–ZnS heterostructures, the p–n junction formed near the interface of Cu_1.94_S–ZnS locates the conduction band of Cu_1.94_S above that of ZnS. The protons are reduced to hydrogen by the electron–hole pairs generated in the semiconductors during irradiation. Thus, the complex heterostructured NCs provide better catalytic activity than their individual components. As shown in Figure 10A(b), the Cu_1.94_S–ZnS heterostructure had a linear increase in hydrogen production with irradiation time, indicating the best photocatalytic hydrogen production with an evolution rate of 0.918 mmol h^−1^ g^−1^, which is 38 and 17 times greater than the individual NCs Cu_1.94_S and ZnS, respectively (Figure 10A(c)). With a hydrogen evolution efficiency of 878.1 μmol h^−1^ g^−1^, core/shell Cu_1.94_S/MnS heterostructures also serve as efficient noble‐metal‐free photocatalysts.^[^ ^55^ ^]^ They exhibit excellent stability by retaining a hydrogen evolution rate of 80% to the initial value after five cycles. In addition, they did not exhibit phase transformation, indicating excellent recyclability for long‐term use. The synergistic effect between the Au and Cds in a Au@CdS yolk–shell nanostructure enables them to pose higher photocatalytic conversion.^[^ ^59^ ^]^ The yolk–shell structures comprising a movable core and an external hollow shell have a higher surface area and smaller density, improving the distribution and number of active sites, thereby enhancing their ability to absorb organic molecules. With the same mass, the surface area of Au@CdS yolk–shell nanostructures was twice that of their core–shell counterparts, exhibiting strong light absorption capacity. These Au@CdS yolk–shell nanostructures could effectively decompose the rhodamine 6G (R6G) solution as compared with CdS nanoparticles and Au@CdS yolk–shell nanostructures.

By sulfidation and kinetically controlled CER in Au/Cu_2_O core/shell NC template, four different yolk–shell NCs such as Au@Cu_7_S_4_, Au@ZnS, Au@CdS, and Au@Ni_3_S_4_ were produced.^[^ ^61^ ^]^ The X‐ray photoelectron spectroscopy (XPS) and PL spectroscopy results showed that there is pronounced charge carrier separation due to the interfacial charge transfer between the semiconductor shell and yolk, making them suitable for photocatalytic applications. The photocorrosion stability issues observed in CdS/Cu_2−*x*_S heterostructure can be improved by fabricating stratified CdS–Cu_2−*x*_S/MoS_2_ heterostructures (Figure 10B(a,b)).^[^ ^60^ ^]^ The Cu^+^ ions migrate and intercalate with the MoS_2_ basal planes exhibiting improved stability and catalytic activity. CdS–Cu_2−*x*_S/MoS_2_ exhibits a photocurrent density of 380 μA cm^−2^, while the core/shell CdS–Cu_2−*x*_S and pristine CdS exhibit a photocurrent density of 20 and 6 μA cm^−2^, respectively. The excited‐state lifetimes of stratified CdS–Cu_2−*x*_S/MoS_2_ dramatically decreased because MoS_2_ behaved as a shunt for charge recombination, suggesting that electrons are concentrated in MoS_2_ (electron acceptance from Cu_2−*x*_S) and an abundance of holes are concentrated in the valance band of Cu_2−*x*_S (hole acceptance from both CdS and MoS_2_) (Figure 10B(c,d)). Therefore, the stratified CdS–Cu_2−*x*_S/MoS_2_ heterostructures achieved a hydrogen evolution rate of 14184.8 μmol g^−1^ h^−1^, 12.7 and 97.2 times greater than the CdS–Cu_2−*x*_S (1121.4 μmol g^−1^ h^−1^) and pristine CdS (146.0 μmol g^−1^ h^−1^), respectively. In addition, Guo et al. reported that by introducing sulfur vacancies in the CdS and subsequently forming core/shell heterostructures with CuS through CERs (CdS–SV@CuS), its photocatalytic activity could be enhanced.^[^ ^62^ ^]^ The desired route for the flow of electrons from the carrier separation sites toward the catalytic sites on the surface is provided by the interfacial charge transfer present at the interface of CdS–SV@CuS core–shell heterostructures. Thus, the electrons trapped in the sulfur vacancies at the CdS coupled with the interfacial charge transfer contribute to the transfer of charges from CdS–SV to CuS for efficient catalytic H_2_ production. Further, using the photoinduced ion‐exchange method, solid‐solution semiconductor heterostructures can be produced, whose bandgap and redox potentials could be tailored to obtain increased photocatalytic activity.^[^ ^63^ ^]^

### 4.3 Electrocatalysis

Electrocatalysts have garnered great attention among researchers with applications in fuel cells and electrolysis. Developing noble‐metal‐free electrocatalysts with low cost and enormous active sites is of prime importance. The composition, defects, specific surface area, active sites, crystal phases, and interfaces of the NCs crucial for electrocatalysis can be tailored using CER. A multitude of transition metal oxides has been used as electrocatalysts. However, their structural variability, multiple valences, the requirement of low reaction temperatures, and difficulty in producing controlled structures with large surface area limit their applications in electrocatalysis.^[^ ^64^, ^65^ ^]^ The regiospecifically designed Mn_3_O_4_@CoMn_2_O_4_ and Mn_3_O_4_@CoMn_2_O_4_–Co_*x*_O_*y*_ core/shell heterostructures with controlled phase distribution and composition exhibited superior activity toward oxygen evolution and reduction reactions as compared with Pt and other reported metal oxides.^[^ ^65^ ^]^ Mn_3_O_4_@CoMn_2_O_4_ was produced by partial cation exchange of Mn^2+^ by Co^2+^ in the existing Mn_3_O_4_ nanoparticle, and with the use of an alternate Co precursor, the surface of Mn_3_O_4_@CoMn_2_O_4_ was nucleated with Co_*x*_O_*y*_ crystallites to form Mn_3_O_4_@CoMn_2_O_4_–Co_*x*_O_*y*_ heterostructures. The electrocatalytic performance can be boosted by enhancing the charge transfer and the number of catalytically active sites. In this regard, cation exchange and the decoration of nanocrystallites on the surface provide increased active sites with low onset potentials. With the molar ratios of Co:Mn = 1, Mn_3_O_4_@CoMn_2_O_4_–Co_*x*_O_*y*_ heterostructures exhibited low overpotentials and Tafel slope of 0.31 V at −3 mA cm^−2^ and 52 mV dec^−1^, respectively, for oxygen reduction reaction (ORR) and with respective values of 0.31 V at 10 mA cm^−2^ and a Tafel slope of 81 mV dec^−1^ for oxygen evolution reaction (OER).

**Figure 11:**
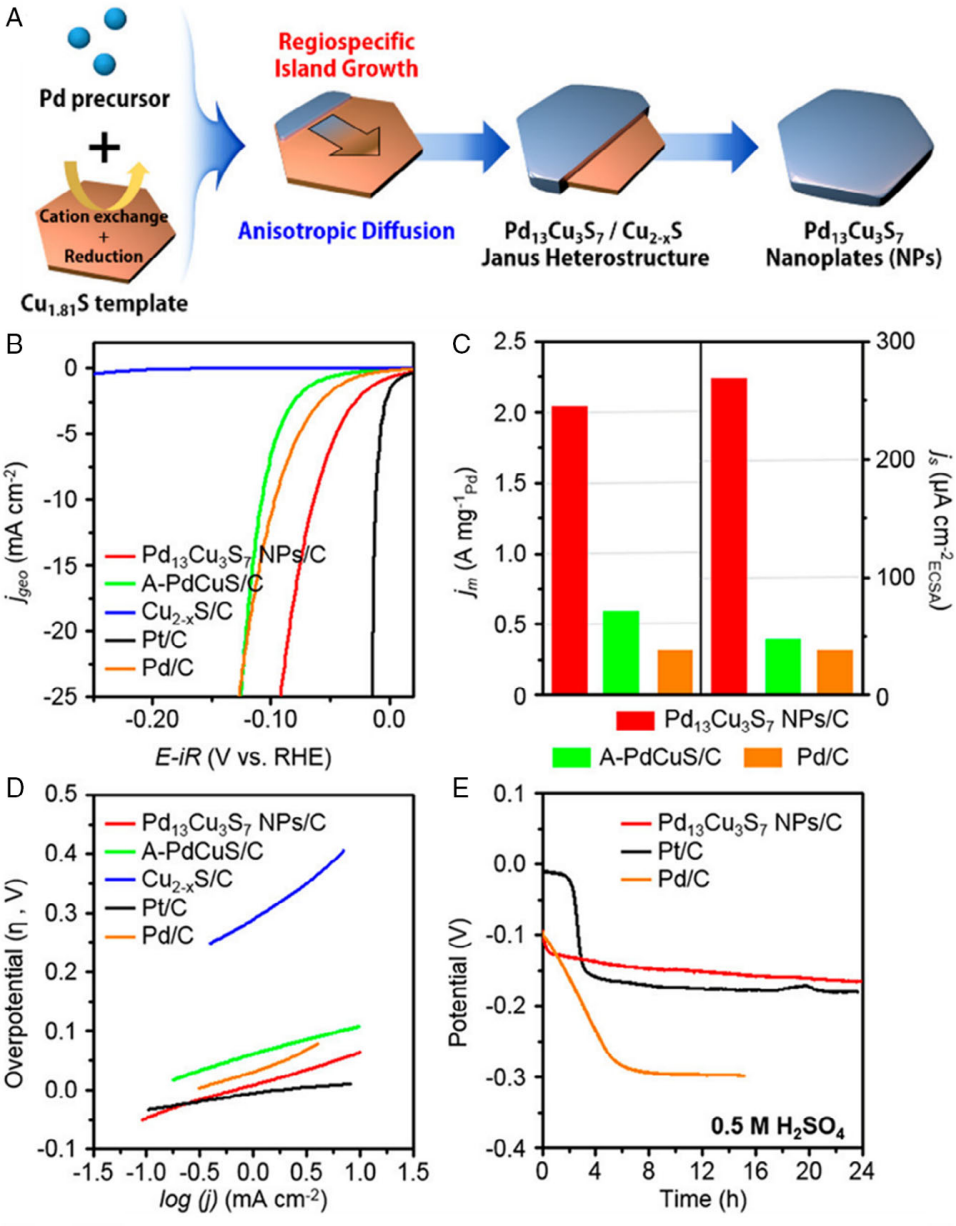
A) Schematic illustrating the synthesis of Pd_13_Cu_3_S_7_ nanoplates from Cu_1.81_S template. B) Polarization curves of different catalysts. C) Comparison of mass and specific activities (*j*_m_ and *j*_s_). D) Tafel plots and E) potential−time curves at a current density of 10 mA cm^−2^. A–E) Reproduced with permission.^[^ ^18^ ^]^ Copyright 2018, American Chemical Society.

Park et al. demonstrated the importance of mixed crystalline phases in enhancing the hydrogen evolution reaction (HER) activity and stability.^[^ ^18^ ^]^ Pd_13_Cu_3_S_7_ nanoplates and the intermediate Janus Pd_13_Cu_3_S_7_/Cu_2−*x*_S heterostructure were produced through partial CERs with Cu_1.81_S hexagonal nanoparticles. The formation of the Pd_13_Cu_3_S_7_ nanoplates was initiated at the edge sites forming Janus‐like intermediates (**Figure** **11**A). The Pd_13_Cu_3_S_7_/C nanoplates had higher mass activity per mass of Pd. They exhibited an excellent HER performance with an overpotential of 64 mV to reach 10 mA cm^−2^, which was significantly lesser than the commercial Pd/C and amorphous PdCuS (Figure 11B–D). This performance indicates the importance of crystalline facets and synergy between the bimetals. Furthermore, Pd_13_Cu_3_S_7_/C produced 8 mL of H_2_ gas, showing nearly 100% Faradaic efficiency for HER with good catalytic stability. Due to the synergistic effects between different metal atoms, binary metal selenides obtained as an intermediate during CER exhibited better OER activity than their monometallic counterparts.^[^ ^66^ ^]^ The slow partial CER between berzelianite Cu_2−*x*_Se (ber‐Cu_2−*x*_Se) and Ni^2+^/Ni^3+^ cations led to the formation of ber‐Cu_2−*x*_Se /spinel Ni_3_Se_4_ heterostructure with immiscible phases and large lattice mismatch. The Janus heterostructure was an active OER catalyst with a low overpotential of 230 mV at 10 mA cm^−2^ in an aqueous 0.1 m KOH. The individual NC phases in the heterostructure synergistically helped to increase the electrochemically active surface area and generated highly active Cu species, resulting in improved performance than the sp‐Ni_3_Se_4_ NCs. Very recently, Wu et al. demonstrated the cation exchange between M^δ+^ (Fe^2+^, Ce^3+^, Fe^3+^, etc.) and MnO NCs covered by a thin Mn_3_O_4_ layer.^[^ ^67^ ^]^ The cations in the Mn_3_O_4_ were exchanged with the M^δ+^ cations without affecting the MnO core. The MnO core was later etched to release the strain between the core and Mn_3−*x*_M_*x*_O_4_ shell. With Fe^2+^ ions, the resultant Mn_3−*x*_Fe_*x*_O_4_ hollow NCs exhibited excellent OER performance with a low Tafel slope of 43.3 mV dec^−1^, large electrochemical surface area, and low charge transfer resistance.

### 4.4 Other Applications

Apart from the above‐discussed applications, the well‐defined heterostructures find applications in energy storage, biomedical, solar cells, photoelectronic devices, sensors, etc., with their unique characteristics. Li et al. reported the bioapplication of core–shell MnS@Bi_2_S_3_ heterostructure coated with poly(ethylene glycol), produced through the CERs.^[^ ^68^ ^]^ The heterostructure served as an efficient diagnostic and therapeutic agent with the Bi_2_S_3_ shell, exhibiting strong X‐ray absorption and broad absorption in the NIR window, indicating their potential use in photoacoustic imaging, computed tomography imaging, radiotherapy, and photothermal therapy. Mesoporous hollow Sb/ZnS@C core–shell heterostructures when used as Na‐ion battery anodes exhibited excellent storage properties.^[^ ^69^ ^]^ The synergistic effects between the components and their unique architectures enable them to pose high reversible capacity, good cycling stability, and rate capability. Wang et al. reported the transformation of binary Bi_2_S_3_ nanorods to ternary AgBiS_2_ nanorods through partial cation exchange, which initiated at the ends of the nanorod due to the presence of high‐density surface dangling bonds and imperfect passivation of ligands at the two ends, making it more reactive than the midpart.^[^ ^70^ ^]^ The ternary AgBiS_2_ nanorods had a bandgap of ≈0.86 eV, which is smaller than the reported AgBiS_2_ nanomaterials. The photocurrent‐to‐dark current ratio at a bias of 1 V is >10 with the rise and decay times for photoresponse being <0.2 s. The ternary AgBiS_2_ nanorods are thus suitable to be employed in photoelectronic applications. A formamidinium (FA)‐based perovskite Cs_0.15_FA_0.85_PbI_3_/Cs_*x*_FA_1−*x*_PbI_3_ core–shell heterostructure with Cs‐rich shells helps to increase the defect formation energy, thereby reducing the defect density.^[^ ^71^ ^]^ A perovskite solar cell constructed with this heterostructure exhibited an efficiency of 20.7% and maintained 95% of its initial efficiency after 1000 h, corresponding to a lifetime of 2 years. Further, the solar cell can operate with the best stabilities at 60 °C (for 250 h) and 85 °C (for 3000 min). Co_3_O_4_/CoWO_4_ core–shell urchin‐like microspheres obtained through ion‐exchange reactions exhibit acetone gas‐sensing properties, indicating their potential to be used as acetone gas sensors.^[^ ^72^ ^]^ Owing to their structural benefits, the fabricated sensor exhibited high response, selectivity, low operating temperature, and excellent sensing stability to acetone vapors. Compared with pristine Co_3_O_4_, the heterojunction interface enabled the formation of different dangling bonds due to the different lattice parameters of Co_3_O_4_ and CoWO_4_, which is beneficial for the adsorption of gas molecules. A response value of 9.9–20 ppm acetone was recorded by the Co_3_O_4_/CoWO_4_ sensor with a response and recovery time of 22 and 12 s, respectively.

## 5 Conclusion and Outlook

There lies a great impetus to impose the regiospecific compositions within the morphology‐defined NCs, because regiospecifically designed heterostructures might exhibit unconventional optical, photocatalytic, and electrocatalytic properties. Compared with the galvanic replacement reactions (GRRs),^[^ ^73^ ^]^ CER demonstrates better ability in controlling the compositional and morphological complexity in heterostructured NCs, aided by a comprehensive understanding of the thermodynamic, kinetics, and a wide selection of crystalline templates. The most notable trend in CERs has been the regiospecific reactions, that is, partial phase transformation, within the morphology‐controlled NCs. In this perspective, we outlined recent advances in CERs, expounding critical factors controlling regiospecificity based on thermodynamics, reaction kinetics, as well as crystal lattice access engineering.(see **Table** **3**) We also reviewed the applications of such regiospecifically transformed heterostructures. Although regiospecificity‐engineered CERs have been developing rapidly in recent years, formidable challenges still exist in the path to the full‐throttled explosive adoption of regiospecific cation‐exchange chemistry in the nanomaterial community. The critical issues remain to be addressed.

**Table 3:** Summary of the regiospecificity‐controlled NCs through CERs

Initial phase	Initial morphology	Exchange element	Final phase	Final morphology	Regiospecificity determinant factor	Exchange reaction method				Application	References
						Solvent	Precursors	Temp. [°C]	Time [min]		
Cu_1.81_S	Plate	Ag^+^	Cu_1.94_S/Ag_2_S	Janus plate	Crystal phase/lattice mismatchk)	OAma)	AgNO_3_	50	30	−	[23]
Cu_2−*x*_Se	Sphere	Zn^2+^	ZnSe–Cu_2_Se	Janus sphere	Crystal phase/lattice mismatch	THFb),Tolc),TOPd)	Zn(NO_3_)_2_	RT	Overnight	−	[24]
Cu_2−*x*_Se	Sphere	Cd^2+^	CdSe–Cu_2_Se	Janus sphere	Crystal phase/ vacancy/lattice mismatch	THF,Tol,TOP	Cd(NO_3_)_2_	RT	Overnight	−	[24]
Cu_2−*x*_Se	Sphere	Zn^2+^	ZnSe–Cu_2_Se	Janus sphere	Crystal phase/ vacancy/lattice mismatch	Tol,OAm, OcAme), TOP,ODEf)	Zn(OAc)_2_ g)	150	1–10	−	[24]
Cu_2−*x*_Se	Sphere	Cd^2+^	CdSe–Cu_2_Se	Janus sphere	Crystal phase/ vacancy/lattice mismatch	Tol,TOP,OAh), ODE	CdO	150	1–10	−	[24]
Cu_2−*x*_Se/Cu_2−*x*_S	Core/shell	Ag^+^	Ag_1.6_Se/Cu_1.8_S	Core/shell	Thermodynamicsl)/ vacancy	MeOH,Tol	AgNO_3_	RT	1	−	[21]
Cu_2−*x*_Se/Cu_2−*x*_S	Core/shell	Hg^2+^	Hg_0.9_Se/Cu_2_S	Core/shell	Thermodynamics/ vacancy	MeOH,Tol	HgBr_2_	RT	1	−	[21]
Cu_2−*x*_Se/Cu_2−*x*_S	Core/shell	Au^3+^	Au_0.7_Se/Cu_2.1_S	Core/shell	Thermodynamics/ vacancy	MeOH,Tol	CuCl_3_	RT	1	−	[21]
Cu_2−*x*_S	Rod	Zn^2+^/ In^3+^/ Ga^3+^/ Co^2+^/ Cd^2+^	ZnS–CuInS_2_–CuGaS_2_–CoS–(CdS–Cu_1.8_S)	HSi) rod	Crystal phase/lattice mismatch	Benzyl ether,OAm,ODE	ZnCl_2_/ InCl_3_/ GaCl_3_/ CoCl_2_/ CdCl_2_	75–130	30–60	−	[7]
Cu_1.8_S	plate	Co^2+^	Cu_1.8_S/w–CoS	Janus plate	Crystal phase/surface energym)	OAm,Tol,TOP	CoCl_2_	100	< 5	−	[44]
Cu_1.8_S	Rod	Co^2+^	Cu_1.8_S/Co_9_S_8_	Janus rod	Crystal phase/surface energym)	OAm,Tol,TOP	CoCl_2_	100	< 5		[44]
Cu_1.8_S	Sphere	Cd^2+^	CdS–CuS	sandwiched sphere	Crystal phase/lattice mismatch	OAm,TOP,ODE	CdCl_2_	100	2	−	[15]
CdS	Sphere	Pb^2+^	PbS–CdS	Janus sphere	Crystal phase/lattice mismatch	OAm,Tol, ODE	PbCl_2_	80–190	< 20	−	[54]
CdSe@CdS	Core/shell rod	Cu^+^	CdSe@CdS/Cu_2−*x*_S	Core/shell rod	Crystal phase/lattice mismatch	MeOHj),Tol	[Cu(CH_3_CN)_4_]PF_6_	RT	5	−	[53]
CdSe@CdS/Cu_2−*x*_S	Core/shell rod	Zn^2+^	CdSe@Cds/ZnS	Core/shell rod	Crystal phase/lattice mismatch	OAm,ODE	ZnCl_2_	250	3	−	[53]
Cu_3−*x*_P	Plate	In^3+^	Cu_3−*x*_P/InP	HS plate	surface energym)/crystal phase/lattice mismatch	TOP,ODE	InBr_3_	200	< 5	NIR Plasmonics	[14]
Cu_1.8_S	Sphere	Zn^2+^	ZnS–CuS	sandwiched sphere	Lattice mismatch/crystal phase	OAm,Tol,TOP	ZnCl_2_	100	10	Surface Plasmon Resonance	[15]
CdSe	Ring	Pb^2+^	PbSe	Ring	Surface energy	OAm,ODE	PbBr_2_	80	420	Photoluminescence quantum ring	[16]
Cu_1.94_S	Sphere	Zn^2+^	Cu_1.94_S–ZnS	Janus sphere	Crystal phase/lattice mismatch	OAm,ODE	ZnCl_2_	105	15	Photocatalytic hydrogen evolution	[17]
Cu_1.81_S	Plate	Pd^2+^	Pd_13_Cu_3_S_7_	Janus plate	Crystal phase/surface energy	OAm	Pd(acac)_2_	160	30	Electrocatalytic hydrogen evolution	[18]

a) OAm: oleyamine;

b) THF: Tetrahydrofuran;

c) Tol: toluene;

d) TOP: trioctylphophine;

e) OcAm: octylamine;

f) ODE: 1−octadecene;

g) OAc: acetate;

h) OA: oleyic acid;

i) HS: heterostructured;

j) MeOH: methanol;

k) Lattice mismatch: Regiospecific CER occurs along certain crystallographic directions with minimal lattice mismatches between the template and resulting phases;

l) Thermodynamics: Morphology and phase determined toward thermodynamically favored states;

m) Surface energy: Regiospecific CER initiated from the specific sites with high surface energy, such as edges, corners of templates.

### 5.1 Dearth of Template Materials

The identity of the inorganic compound template is utterly important to the fate of CERs, because the thermodynamic and kinetic control completely depend on ion mobility and the direction of ion infusion within the template, which determines the compositional and morphological complexity of CERs. Due to the high mobility of copper ions and thus the ease of cation exchange with other metal ions, most cation exchange studies have utilized copper‐based chalcogenide materials, and most regiospecific heterostructures are also based on the 0D copper sulfide NCs.^[^ ^7^, ^11^, ^74^ ^]^ The availability of synthetic routes in mass‐producible copper sulfide systems also helped the concentrated work with only copper sulfide nanotemplates. For example, highly monodispersed 2D Cu_1.94_S (djurleite) hexagonal nanoplates can be produced via a facile one‐pot scalable synthetic route using CuSCN single precursor,^[^ ^2^ ^]^ and thickness‐controlled 1D Cu_1.8_S NCs could be synthesized by modulating the stoichiometry between CuCl_2_ and Cu(NO_3_)_2_ precursors.^[^ ^75^ ^]^ On the other hand, the high mobility of copper ions might be reproduced in nonsulfide copper compound systems. For example, Hong et al. showed the versatility of a copper phosphosulfide hollow toroid as a cation‐exchange template for the synthesis of various transition metal phosphosulfides.^[^ ^76^ ^]^ Therefore, it would be advantageous to develop anion‐exchange routes applicable to copper sulfide systems to exploit the high mobility of copper ions as well as to benefit from the mass production ability of the starting copper sulfide nanoparticles; anions of P, Se, and Te atoms might be particularly useful. In addition to the derivative formation from copper sulfides, direct mass production of copper compounds of P, Se, Te, and other anions should be attempted for full‐throttled adoption of cation‐exchange chemistry. The anion‐engineered system can realize completely different types of structures due to the presence of potential Kirkendall effect during the process. Diversification of anion composition can also exponentially expand the present megalibrary diversity realized by CERs. Moreover, the multianion system has verified its effectiveness in various fields such as batteries, optics, and electrocatalysts, showing superior electrochemical and optical properties.^[^ ^77^, ^78^ ^]^ Consequently, in parallel with efforts to study CERs, future research to develop advanced nanomaterials with much improved physicochemical properties should be extended to create anion‐engineered CER templates.

In addition, the highly focused work on copper‐based templates is due to the easy oxidation change of copper cation and facile formation of vacant sites in the crystal lattice, which is hard to find in other metal cations. Nonetheless, other cation systems are also amenable to cation exchange with proper amounts of vacancies and anion framework structures. Therefore, it is necessary to explore the availability of cation‐exchange pairs in other cation systems. It would also be profitable to explore ternary or more complex systems containing copper cations because such systems would inherit the rampant formation of vacant sites and thus ease the cation exchange of copper‐only systems.

### 5.2 In‐Depth Understanding of the Crystallographic Features

Cation exchange relies heavily on the openness of the anion framework, as demonstrated by the access‐limited cation exchange on the HCP Cu_1.81_S template covered with face‐centered‐cubic (FCC) Ir_*x*_S_*y*_ crown.^[^ ^50^ ^]^ The unprecedented CER was achieved by understanding the anion framework feature of FCC Ir_*x*_S_*y*_ and HCP Cu_1.81_S, which allowed anisotropic Au^+^ and Pd^2+^ cation entry sites on HCP Cu_1.81_S. The lattice mismatches between the resulting Au_2_S, PdS, and Cu_1.81_S produced different Janus heterostructures. Also, the number of vacant sites and ease in the cation diffusion, affected by the oxidation switching ability of cations and Coulombic attraction between cation and anion, respectively, gravely determine the direction of cation entry as well as the overall success of cation exchange. Therefore, it is utterly necessary to fully understand the crystallographic feature of the template prior to designing a CER. Furthermore, the lattice parameters of facets at juxtaposed heterointerfaces play a pivotal role in determining the degree of surface strain or the electron transfer efficiency during the catalytic reaction, which is a major application area of the heterostructures. The similarity of the anion sublattice of the original template to that of the final structure would guarantee the success of cation exchange. However, the notable differences in the anion framework at the interface and the resulting interfacial strain are also highly desired for successful application in catalysis. In addition, it has been reported that the synthesis of hetero‐nanostructures with large lattice mismatches was also achievable through delicate control of CER, which can also lead to complex hybrid nanostructures.^[^ ^79^, ^80^, ^81^, ^82^, ^83^, ^84^ ^]^ Therefore, the compromise between the ease of cation exchange and the degree of interfacial strain should be carefully met and be considered prior to the actual synthetic works, which requires the development of theoretical tools that predict the feasibility of cation exchange along certain crystallographic directions.

### 5.3 Crystal Phase Stability Issue in Practical Applications

Compositionally and morphologically well‐defined NCs have been designed and synthesized for various applications. However, the phase stability of kinetically approached regiospecificity‐controlled NCs must be carefully considered before attempting to use them in practical applications. Recently, Li et al. reported that the presence of thermodynamically unstable exposed facets at the template material or the significant volume change between starting and final phases during the CER could induce drastic phase transformation to a stable phase by reconstructing the anion sublattice.^[^ ^44^ ^]^ As the resulting heterostructured NCs should maintain their original structures with promising properties without changing both phase and morphology, tortuous synthetic works without considering the phase stability can lead to only undesirable application aspects, which should be avoided at all costs.

In summary, there are still many formidable hurdles to the full expansion of regiospecificity‐controlled cation exchange chemistry. However, it should be emphasized that the potential of this branch of chemistry for various application fields is irrefutable. We hope that this perspective has properly highlighted new research avenues to unconventional heterostructures with high compositional and morphological diversities and also with fitting application prospects through a simple but carefully designed cation‐exchange chemistry.

## Conflict of Interest

The authors declare no conflict of interest.
